# Evaluation on the Efficacy and Safety of Panax Notoginseng Saponins in the Treatment of Stroke among Elderly People: A Systematic Review and Meta-Analysis of 206 Randomized Controlled Trials

**DOI:** 10.1155/2023/4312489

**Published:** 2023-05-04

**Authors:** Peiyu Guan, Dingkun Gui, Youhua Xu

**Affiliations:** ^1^Faculty of Chinese Medicine, State Key Laboratory of Quality Research in Chinese Medicines, Macau University of Science and Technology, Taipa, Macao, China; ^2^Department of Nephrology, Shanghai Jiao Tong University Affiliated Sixth People's Hospital, Shanghai, China; ^3^Macau University of Science and Technology Zhuhai MUST Science and Technology Research Institute, Hengqin, Zhuhai, China; ^4^Zhuhai Hospital of Integrated Traditional Chinese and Western Medicine, Zhuhai, China

## Abstract

**Background:**

Evidence regarding the effect of Panax notoginseng saponins (PNS) on treating elderly stroke patients is scare and inconsistent. This study investigated the efficacy and safety of PNS by means of meta-analysis so as to provide an evidence-based reference for the treatment of elderly patients with stroke.

**Methods:**

We searched the PubMed, Embase, Cochrane Library, Web of Science, CNKI, VIP, Wanfang, and China Biomedical Database to identify the eligible randomized controlled trials (RCTs) concerning using PNS to treat elderly people with stroke from their inception to first, May 2022. Meta-analysis was used for pool analysis of the included studies, whose quality was assessed via Cochrane Collaboration's RCT risk of bias tool.

**Results:**

Altogether 206 studies published between 1999 and 2022 with a low risk of bias were included, covering 21,759 participants. The results showed that the improved neurological status shown in the intervention group with PNS alone was statistically significant (SMD = −0.826, 95% CI: −0.946 to −0.707) in contrast to the control group. The total clinical efficacy (Relative risk (RR) = 1.197, 95% Confidence interval (CI): 1.165 to 1.229) and daily living activities (SMD = 1.675, 95% C: 1.218 to 2.133) of elderly stroke patients were significantly improved as well. In addition, the invention group using PNS combined with WM/TAU displayed significant improvement in neurological status (SMD = −1.142, 95% CI: −1.295 to −0.990) and the total clinical efficacy (RR = 1.191, 95% CI: 1.165 to 1.217) compared with the control group.

**Conclusion:**

Single PNS intervention or PNS combined with WM/TAU significantly improves the neurological status, the overall clinical efficacy and daily living activities of elderly stroke patients. However, more multicenter RCT research with high quality is required in the future to verify the results in this study. The trial registration number: Inplasy protocol 202330042. doi:10.37766/inplasy2023.3.0042.

## 1. Introduction

Stroke, a major cause of disability and death, is a common disease among elderly people. As the second cause of death among people aged over 60 around the world, its mortality is on the rise every year. China has the highest incidence of stroke, where 1,763 out of 100,000 people suffer from ischemic stroke per year [1]. Meanwhile, disability rate of stroke is as high as 75% in China [2]. According to a report from Global Burden of Disease, 1.7 million people died from stroke in 2010 [3]. In recent years, stroke has become the first cause of death in China [4]. According to statistics, from 2015 to 2018, around 2% of people over 40 years old had a stroke, up to 50% of whom were no more than 64, which suggest that the average age of stroke onset is going down. With a high rate of prevalence, disability and mortality, stroke has thus become a global public health concern.

Internationally, the main treatment adopted now for stroke is still vascular recanalization (thrombolysis and endovascular interventional therapy), whose effect [5, 6], however, is enjoyed by only a few patients due to factors such as short time window, high cost, and limited medical level. Panax notoginseng saponins (PNS), with the functions of dispersing blood stasis and hemostasis, reducing swelling and relieving pain, is widely used for treating stroke in China. Modern pharmacological studies believe that PNS can reduce the infarct size of ischemic stroke, inhibit edema [7], protect the blood-brain barrier, reduce nerve damage, and inhibit relevant inflammation [8, 9]. Commonly used PNS products mainly include oral-type products, such as Xueshuantong capsule (XC), Xuesaitong soft capsule (XSC), Sanqi Tongshu capsule (STC), and compound Xueshuantong capsule (CXC), and injection type products, such as Xueshuantong capsule, Xuesaitong soft capsule, Sanqi Tongshu capsule, and compound Xueshuantong capsule.

With the increase of age, the elderly are more susceptible to stroke hemiplegia because of gradually declining body function and continually weakening cardiovascular system. For these patients, Western medicine (WM) often adopts recovery treatment that helps improve movement function to a certain extent, but rehabilitation cannot be achieved [10]. Although PNS has been extensively used for treating stroke and many clinical trials have confirmed its clinical efficacy and safety [11, 12], whether it is effective and safe for the elderly population remains to be investigated. However, related evidence-based studies for this age group were rarely reported and the majority of previous studies measured only a single stroke outcome (eg., clinical efficacy). Furthermore, there were many problems with the methodological quality of previous studies. For instance, the studies included had low quality and serious bias, which may adversely affect the credibility of evidence and confuse subsequent clinical practice and health decision-making. As a result, this study, targeting at the elderly population, comprehensively analyzed the efficacy and safety of PNS in multiple outcomes by meta-analysis in order to provide more systematic clinical evidence for clinical medication and health decision-making concerning elderly stroke patients.

## 2. Methods

This study was reported in strict accordance with the Priority Reporting Item for Systematic Reviews and Meta-Analysis (PRISMA) [13]. All the analyses were based on previously published studies, and therefore, no ethical approval and participants' consent were required.

### 2.1. Search Strategies and Study Selection

Based on the standard of the Cochrane Collaboration, a comprehensive literature search, without restrictions on publication time, literature type, or region, was conducted to identify randomized controlled trials (RCTs) related to treating elderly stroke patients with PNS from their inception to first, May 2022, in PubMed, Embase, Cochrane library, Web of Science, CNKI, VIP, Wanfang, and China Biomedical Database. References in the included studies, related conference abstracts, published research papers and gray literature in the form of government reports, etc., are all consulted in case of leaving out any potentially useful data. The literature search was performed based on the combination of subject words and free words. The Chinese search terms included stroke, cerebral infarction, cerebral embolism, cerebral apoplexy, ischemic stroke, ischemic stroke, Panax notoginseng saponins, blood embolism Tong, Xueshuantong, Sanqi Tongshu Capsules, and randomized controlled trials. The English search terms were Xueshuantong capsule, Sanqi Tongshu capsule, Xuesaitong soft capsule, brain infarction, compound Xueshuantong, cerebral infarction, stroke, brain embolism, ischemic stroke, cerebrovascular disorders, and RCT. The specific search strategies of each database were attached in Appendix.

After the initial search, the collected studies were screened to remove duplicates. Ineligible articles were filtered out according to their titles and abstracts. For the remaining potentially relevant results, their full texts were reviewed and assessed according to our screening criteria, during which ineligible articles were excluded, numbered, and then registered with the reasons why they were ruled out. For texts with incomplete information or problems, we evaluated their eligibility after contacting the author.

### 2.2. Inclusion and Exclusion Criteria

In this study, the screening criteria conformed with the PICOS (population, intervention, comparators, outcomes, and study design) principles of the Cochrane Collaboration to assess the quality of studies. Detailed information is listed below.

### 2.3. Population

The symptoms of the population were in line with the relevant diagnostic criteria for stroke both at home and abroad and were confirmed as ischemic stroke by medical imaging tests such as MRI or CT. International criteria formulated by the World Health Organization, the National Center for Neurological Disorders and Stroke Research, and the Japanese Ministry of Health and Welfare were considered. Domestic criteria include ischemic stroke in arteriosclerosis thrombosis cerebral infarction, cerebral embolism, and lacunar cerebral infarction diagnosis standard set by National Cerebrovascular Disease Conference, Stroke Therapeutic Effect Evaluation Standard of Traditional Chinese Medicine (TCM) Diagnosis set by the Chinese Institute of TCM, Tentative Evaluation Standard for Stroke Diagnosis and Therapeutic Effect set by the State Administration of TCM Encephalopathy Emergency research consortium in 1995, Diagnostic Basis, Syndrome Classification and Therapeutic Effect Evaluation of Stroke in The Traditional Chinese Medicine Industry Standard of People's Republic of China—Standard of Diagnosis and Curative effect Evaluation of TCM Disease, and the classification standard in Chinese Classification of Cerebrovascular Diseases 2015 [14]. The subject groups were older adults with an average age of over 60, regardless of gender or race [15].

### 2.4. Intervention

Intervention involves single use of PNS such as Xuesaitong injection, Xueshuantong injection, Lulutong injection, Sanqitongshu capsule, Xuesaitong soft capsule, Xuesaitong Tablets, Xueshuantong Capsules, and Xuesaitong Dropping Pills, combined use of PNS and WM or PNS and treatment as usual (TAU).

### 2.5. Comparator

All the patients in the controlled group underwent conventional routine treatment to improve their cerebral blood supply and drug treatment, such as taking medication to nourish their brain tissues. Conventional therapy in WM, which followed Chinese Guidelines for the Diagnosis and Treatment of Acute Ischemic Stroke 2015 [5], includes intravenous thrombolysis, endovascular therapy, antiplatelet, anticoagulation, decrease of fibrinogen, increase of blood volume, improvement of cerebral circulation, nutrition of nerves, lipid regulation, blood pressure reduction, hypoglycemia, and rehabilitation. The regular treatments for the controlled groups and the treated groups must be the same, and the course of treatment is not limited.

### 2.6. Outcomes

Measurement for the outcomes should be clearly defined and includes at least one of the following items: neurological deficit score, the clinical response rate, and assessment of activities of daily living (ADLs).

### 2.7. Study Design

All the included studies were RCTs or clinical controlled trials. The study design adopted RCT. In other words, “random grouping” should be mentioned in the article or its grouping method was “tossing a coin,” “drawing lots,” “rolling dice,” “random number table,” “computer coding,” “block randomization,” or “stratified randomization.”

### 2.8. Exclusion Criteria

Exclusion criteria were as follows: (1) research with duplicate publications or duplicate data; (2) research with incomplete data or serious errors; (3) research without the full text; (4) research involving unconventional treatments in Western medicine such as Chinese herbs or acupuncture; (5) observational research, fundamental research based on cell or animal specimens, experience summaries, review papers, and case study reports.

### 2.9. Outcome Measures and Data Extraction

The primary outcome measure was the post-treatment neurological deficit score graded by the National Institutes of Health Stroke Scale (NIHSS). Secondary outcome measures included (1) overall clinical response rate [16] and (2) posttreatment ADL score.

The included studies were numbered for the convenience of reviewing. Basic information and data in these studies consisted of title, authors, publication year, specific treatments, number of cases, sample ages, male to female ratio, experimental design methodologies (including randomized method, blind method and the like), key factors for evaluating risk of bias, the outcome measures and the results, the course of treatment, etc.

### 2.10. Quality Assessment of the Included Studies

The risk of bias in the included studies was assessed via the Cochrane Collaboration's RCT risk of bias tool [17]. There were 7 assessed items random sequence generation, allocation concealment, blinding of participants and intervention providers, blinding of outcome assessors, outcome completeness, selective reporting of outcomes, and other sources of bias, which were rated as low, and unclear or high bias level. The assessment of included RCTs was separately conducted by two researchers, who then exchanged the results and checked. Disagreement would first be discussed by the two researchers, who would refer to the supervision researcher if they could not reach a consensus. Finally, the risk of bias map was drawn with RevManand Office software.

### 2.11. Statistical Analyses

Categorical data (such as the overall clinical response rate) were to be combined and measured by relative risk (RR) and numerical data (such as neurological deficit score, and the activity of daily living score) by standard mean difference (SMD), whose 95% of confidence interval (CI) was calculated. The heterogeneity of the included studies was measured by the chi-square test (with a significant level of 0.1) and judged by the value of *I*^2^ at the same time. During the meta-analysis, when statistically significant heterogeneity (*P* < 0.10 or *I*^2^ > 50%) was shown, the random effect model would be chosen; otherwise, (*P* ≥ 0.10 or *I*^2^ ≤ 50%) the fixed effect model would be applied [18]. For significant heterogeneity, subgroup, or sensitivity analysis or only descriptive analysis was conducted to deal with the data. If more than ten studies were concerned with one certain variable, the publication bias would be assessed by a funnel plot and Egger's test [19]. The above data analyses were done with the help of Software STATA (Version 14.0, Stata, Corp, College Station, TX).

## 3. Results

### 3.1. Literature Screening

**Figure 1:**
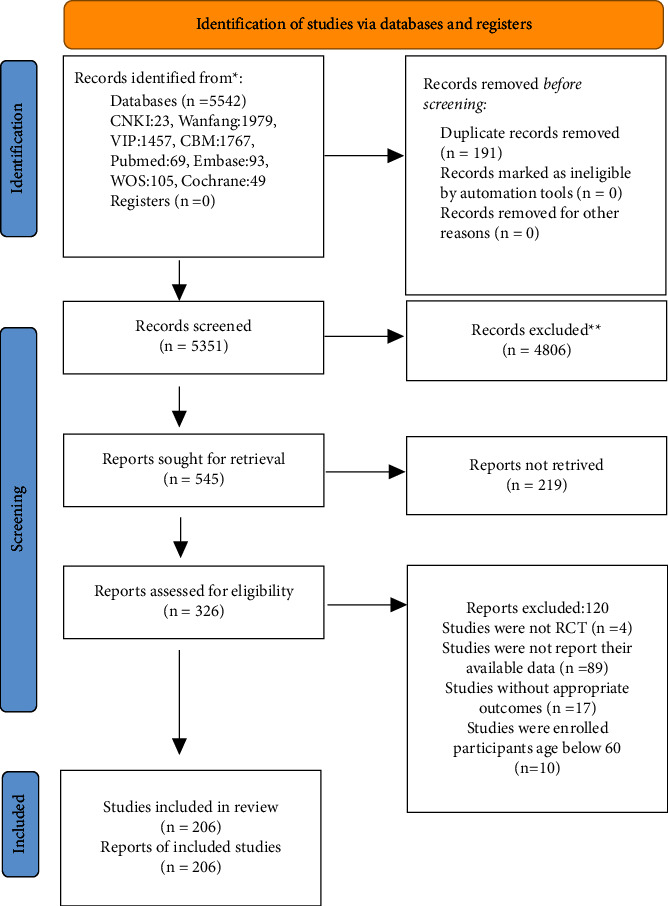
The detailed information on literature screening.

The initial database search yielded 5,542 articles and 5,351 remained after removing duplicates by Endnote. Then, 545 articles were removed by checking titles and abstracts, and 120 articles were excluded from the rest after the full text review. Finally, 206 eligible papers were included for quantitative analysis. The detailed information on literature screening is presented in Figure 1 [13, 20].

### 3.2. Characteristics of the Included Studies

This study collected 206 RCTs for analysis, with 21,759 participants involved, among whom 11,118 were randomly distributed in the intervention group and 10,641 in the controlled group. All included articles were published between 1999 and 2022, 204 of which are Chinese. The average age of participants was 63.20 years old with 12,502 male participants, accounting for 57.46%. The course of treatment varied from 3 days to 6 months. 108 studies reported acute stroke period, 4 studies reported the recovery period, and 94 did not report the information of stroke periods. See Table 1 for all basic information on the included studies.

### 3.3. Quality Assessment

**Figure 2:**
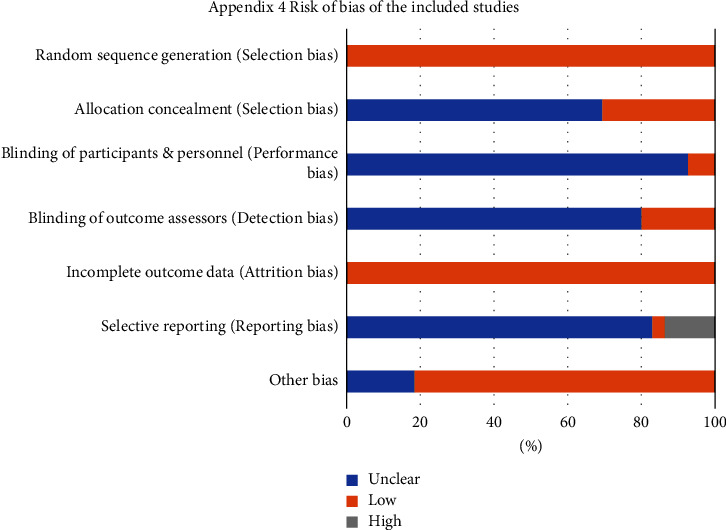
ROB summary (risk of bias of the included studies).

All of the 206 articles were RCTs, which were considered low risk of bias in the generation of random sequences. Without clarifying allocation concealment, 143 articles were rated unclear risk of bias. 15 articles reported blinding of participants and 41 used blinding for assessors. All included studies were at low risk of bias in terms of outcome completeness. 7 articles were rated as low risk for selective reporting bias. For other bias, 38 and 168 studies were at unclear and low risk of bias, separately. Detailed information on the quality assessment of the included studies were introduced in Table 2 and Figure 2. Moreover, certainty assessment of the included studies was shown in Table 3.

### 3.4. Primary Outcome: Neurological Deficit Score

**Figure 3:**
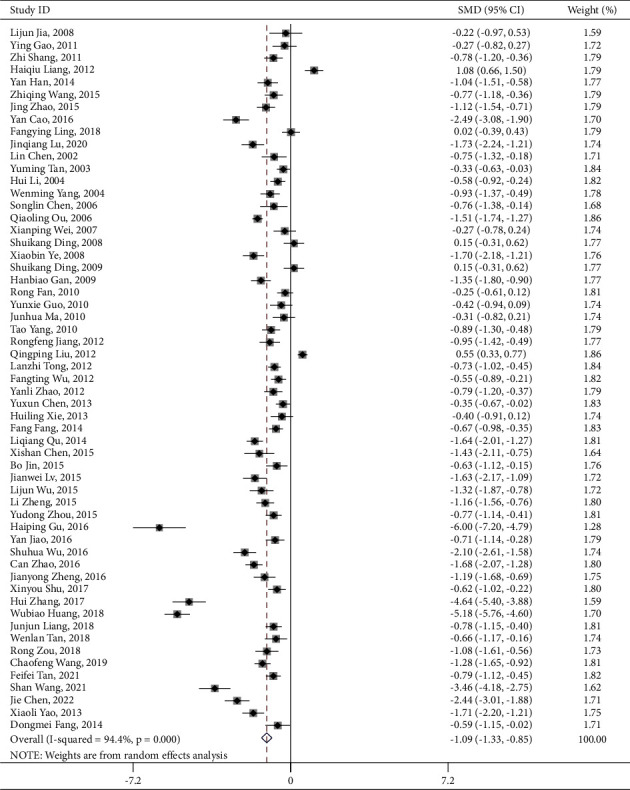
PNS alone NIHSS forest plot.

**Figure 4:**
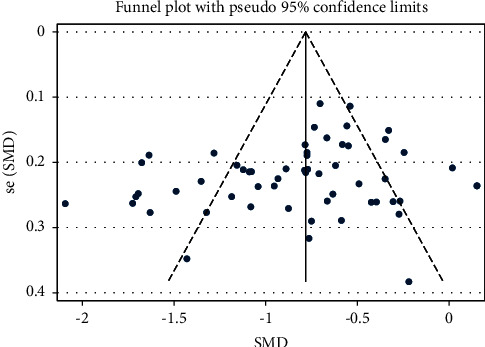
NIHSS-SMD-Ran-funnel plot.

Centered on applying single PNS to treatment, 59 articles involving 6,045 participants reported information on neurological deficit. The intervention group significantly improved the neurological conditions of elderly stroke patients (SMD = −0.826, 95% CI:−0.946 to −0.707; *I*^2^ = 78.9%, *P*_heterogeneity_ < 0.001) (Table 4 and Figure 3) [16]. The funnel plot and Egger's test results indicated no publication bias (*P* = 0.066) (Figure 4).

**Figure 5:**
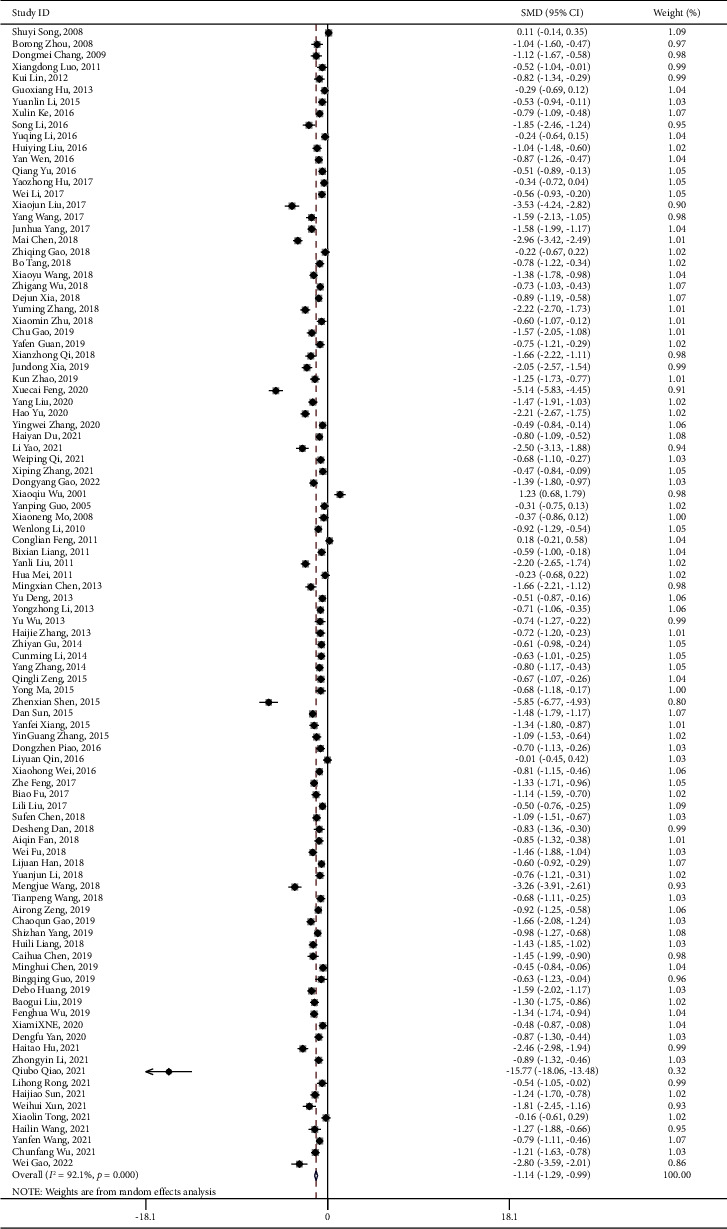
PNS combination NIHSS forest plot.pdf.

**Figure 6:**
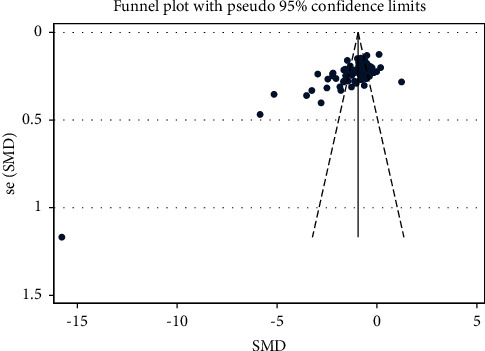
NIHSS-SMD-Ran-funnel plot.pdf.

By contrast, 100 articles involving 10,347 participants reported information on neurological deficit after the treatment of combining PNS with WM/TAU. Similarly, the intervention group showed a significant improvement in the neurological conditions (SMD = −1.142, 95% CI:−1.295 to −0.990; *I*^2^ = 92.1%, *P*_heterogeneity_ < 0.001) (Table 4 and Figure 5) of elderly stroke patients. There was no hint of publication bias in the funnel plot and Egger's test results (*P* < 0.001) (Figure 6).

### 3.5. Secondary Outcomes: Total Clinical Efficacy

**Figure 7:**
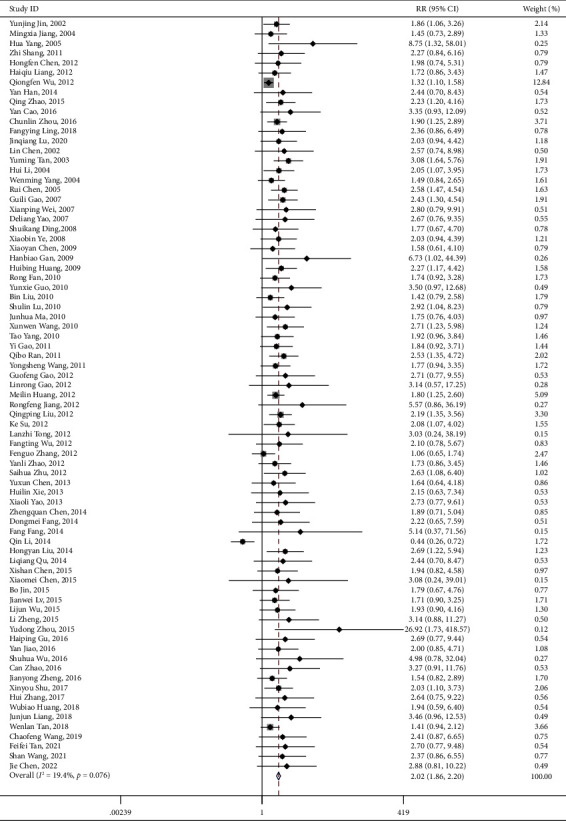
PNS alone total clinical effect forest plot.

**Figure 8:**
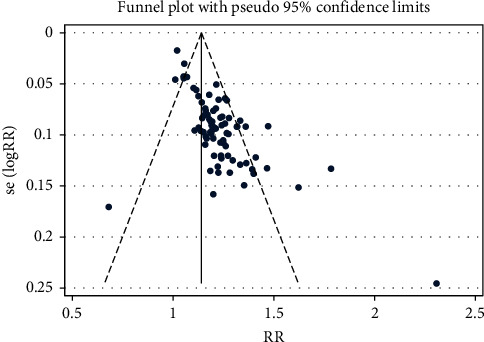
Total clinical effect-RR-Ran-funnel plot.pdf.

There were 77 articles reporting the outcomes of overall clinical efficacy for using PNS alone, involving 8,589 participants. Our results indicated that, compared to the controlled group, the overall clinical efficacy of the PNS alone group showed statistically significant difference (RR = 1.191, 95% CI: 1.165 to 1.217; *I*^2^ = 52.9%, *P*_heterogeneity_<0.001) (Table 4 and Figure 7). The funnel plot and Egger's test results revealed hint of publication bias (*P* < 0.001) (Figure 8).

**Figure 9:**
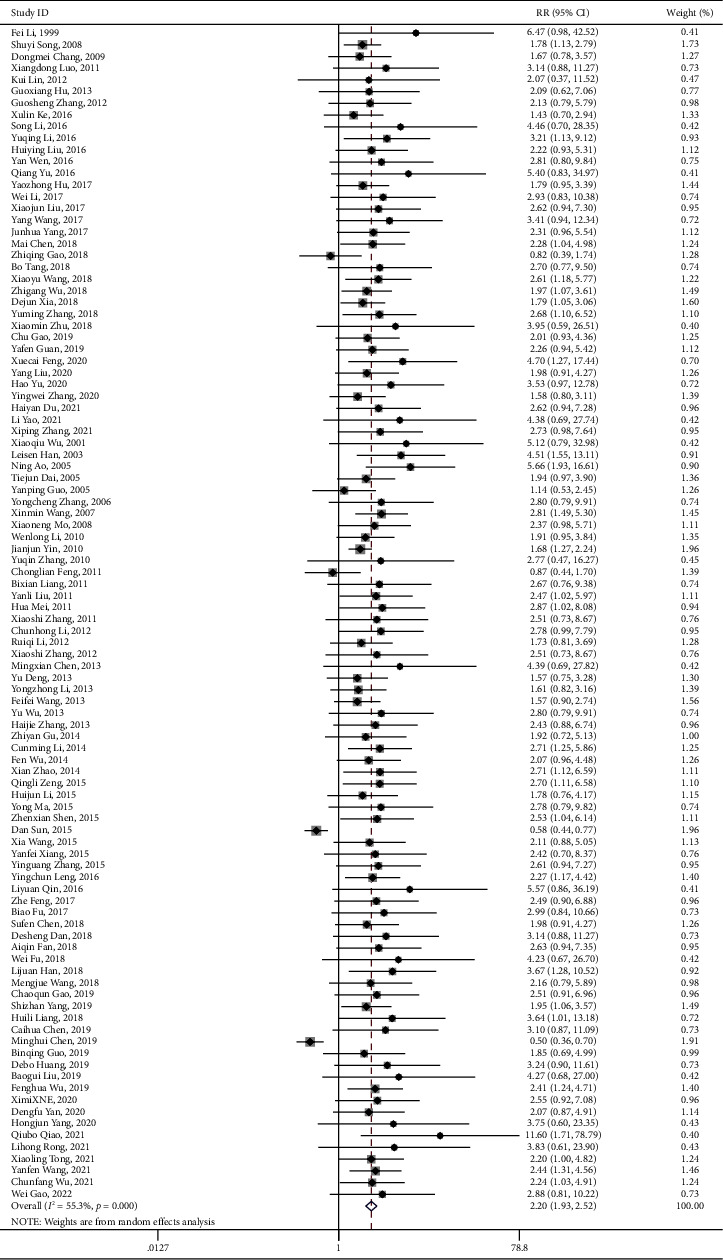
PNS combination total clinical effect forest plot.

**Figure 10:**
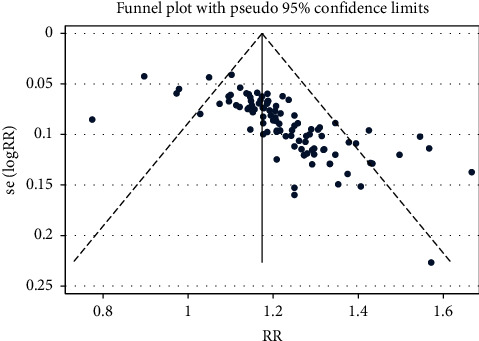
Total clinical effect-RR-Ran-funnel plot.pdf.

For the intervention group using PNS combined with WM/TAU, 100 articles reported the outcome of overall clinical efficacy, with 10,249 participants involved. Compared with the controlled group, there was statistical significance in the overall clinical efficacy of PNS combined with WM/TAU in treating elderly stoke (RR = 1.191, 95% CI: 1.165 to 1.217; *I*^2^ = 42.8%, *P*_heterogeneity_<0.001), as shown in Table 4 and Figure 9. Hint of publication bias was observed in the funnel plot and Egger's test results (*P* < 0.001) (Figure 10).

### 3.6. Activities of Daily Living Score

**Figure 11:**
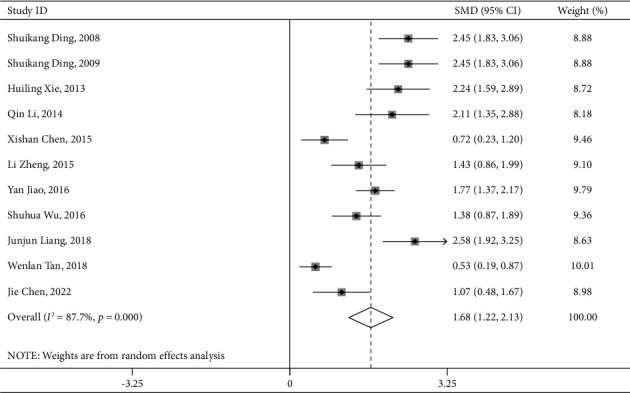
PNS alone ADL forest plot.pdf.

**Figure 12:**
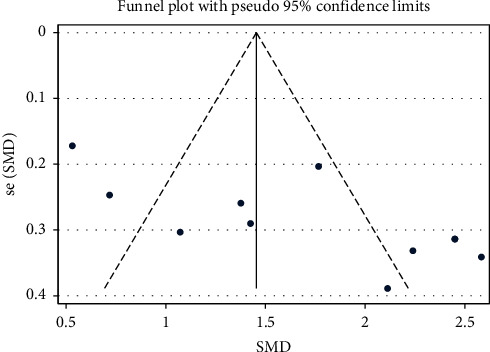
ADL-SMD-Ran-funnel plot [1].pdf.

There were 11 articles reporting ADLs of 839 elderly stroke patients who were treated with PNS alone. As shown in Table 4, elderly stroke patients in the intervention group who were treated with PNS displayed significant improvement in their ADLs (RR = 1.675, 95% CI: 1.218 to 2.133; *I*^2^ = 87.7%, *P*_heterogeneity_<0.001) (Table 4 and Figure 11). The funnel plot and Egger's test results hinted the existence of publication bias (*P* = 0.012) (Figure 12).

**Figure 13:**
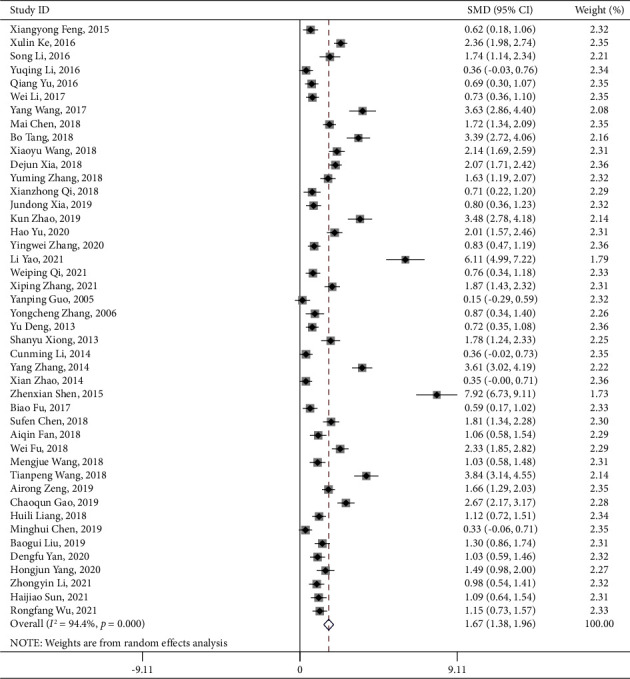
PNS combination ADL forest plot.pdf.

**Figure 14:**
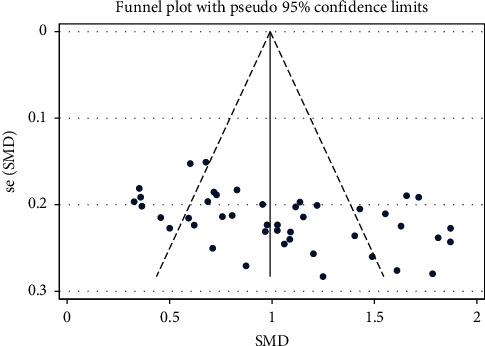
ADL-SMD-Ran-Funnel plot.pdf.

As for the effect of PNS combined with WM/TAU on ADLs in elderly stroke patients (SMD = 1.034, 95% CI: 0.900 to 1.168; *I*^2^ = 77.8%, *P*_heterogeneity_<0.001) (Table 4 and Figure 13), 44 articles with 4,508 participants showed that compared to the controlled group, elderly stroke patients in the intervention group did not improve significantly. The funnel plot and Egger's test results indicated hint of publication bias (*P* = 0.003) (Figure 14).

### 3.7. Subgroup Analysis

Due to the significant heterogeneity of total clinical efficacy, subgroup analysis was conducted based on the following variables: area (developed vs. developing areas), publication year (before vs. and after 2015), sample size (less vs. no less than 100), and male to female ratio (below vs. not below one). The analysis results showed that, for the single PNS intervention, heterogeneity mainly came from articles published before 2015 (Table 4). For the intervention group using PNS combined with WM/TAU, the primary source of heterogeneity was articles with a sample size of less than 100 or with a male to female ratio of less than 1. Results of the analyses revealed that the source of heterogeneity potentially correlated with publication year, treatment duration, and total sample size as well as region development condition. The subgroup analysis results are shown in Table 4.

## 4. Discussion

In this study, 206 articles involving 21,759 participants were collected for meta-analysis. The results showed that compared with the controlled group, single PNS or PNS combined with WM/TAU significantly improved the neurological status, overall clinical efficacy, and ADLs of elderly stroke patients.

In clinical treatment for ischemic stroke, antiplatelet, statin, and antihypertension were the three “cornerstones” [21]. However, the application of WM hit a bottleneck due to issues such as drug resistance, impairment of liver and kidney function, and interactions caused by the co-use of multiple drugs and the like. On the strength of traditional Chinese medicine theory and experience, Chinese medical workers have achieved favorable results in preventing and treating cerebrovascular diseases with natural medicines. After extensively reviewing and analyzing 206 PNS-related clinical trials, this study confirmed the effectiveness of PNS in the treatment of stroke in the elderly population and provided a theoretical basis for treating them in the field of traditional Chinese medicine.

As a traditional Chinese medicine with a history of over 600 years, PNS has the functions of promoting blood circulation, removing blood stasis, reducing swelling, relieving pain, etc. PNS products, such as Sanqitongshu capsule, are mainly composed of panaxtriol saponins (PTS), a component in Sanqi capable of promoting blood circulation and removing blood stasis [22, 23]. By now, the mechanism of PNS is still unclear. It is likely that PTS reduces endothelin levels in the peripheral blood, increases cerebral blood supply to ischemic areas, regulates blood hypercoagulability, and improves the microcirculation in ischemic brain tissue [24]. The major active component in PTS, Rg1 (accounting for 60%), and R1 and Re (accounting for 20%), can improve cerebral ischemic dysfunction, restore ischemic cerebral metabolic abnormalities, resist platelet aggregation, and reduce blood viscosity in the treatment of cerebral arterial thrombosis. In addition, Rg1 enhances the activity of the fibrinolytic system, promotes the release of nitric oxide from the vascular endothelium, and thus, has an antithrombotic effect [25]. For ischemic reperfusion injury, PNS can reduce calcium overload, cerebral edema, and structural damage and promote nerve repair during reperfusion [26] and help reduce mortality during ischemic-reperfusion. What is more, relevant studies show that PNS enhances the ischemic tolerance of the brain and reduces the recurrence of fatal ischemic brain injury, which is beneficial to the secondary prevention of ischemic stroke [27].

PNS has a wide range of pharmacological effects, including scavenging free radicals and antioxidative stress, inhibiting inflammatory factors, blocking calcium ion channels, improving microcirculation and energy metabolism, etc. These effects account for the favorable results of PNS in the treatment of ischemic cerebrovascular disease. PNS can dilate cerebral blood vessels so that cerebrovascular resistance reduces and cerebral blood flow increases. For animals, experimental results indicated that mean blood pressure (BMP) and cerebrovascular resistance (CVR) of anaesthetized rabbits and rats are reduced after the intervention of PNS, depending on the dosage used, but their cerebral blood flow did not increase [28]. PNS for injection, made of Sanqi, has high bioavailability, rapid action, and definite curative effect, which, however, is likely to cause adverse reactions, because it is complicatedly composed, the content of its active components and impurities is difficult to control, and it acts on multiple aspects of the brain. Therefore, it is essential to specify the ingredients and their doses in the injectable PNS so that the quality of the injection as well as the safety of patients can be ensured [28]. Related preparation research explained that compound Xueshuantong capsules effectively reduce blood viscosity, inhibit platelet activation and aggregation, enhance vasodilation regulation function, and increase fibrinase activity, thereby mitigating the cerebrovascular injury caused by oxidative stress response due to ischemic oxygen feeding. Additionally, compound Xueshuantong capsules reduce blood viscosity, regulate blood lipids, and block calcium ion channels, which is conducive to the recovery of cranial nerve function [29–32]. Oral PNS can be used for treating acute stroke by way of multi-target and multi-path. It can improve cerebral blood supply, repair nerve function, narrow the infarct size, and improve the clinical prognosis displaying the clinical advantages of traditional Chinese medicines.

Subgroup analysis discovered that PNS intervention or PNS combined with WM/TAU improved the overall clinical efficacy, neurological status, and ADLs of elderly stroke patients. Sources of heterogeneity probably came from articles with smaller sample sizes or older publications. Conclusions drawn from these articles were less reliable and the evidence was not fully updated, consequently giving rise to heterogeneity in our meta-analysis results.

To our knowledge, this is the first meta-analysis of the clinical efficacy and safety of PNS in the treatment of elderly stroke patients. Based on extensive experimental data, this study made a scientific and objective evaluation of the efficacy and safety of PNS in the treatment of acute elderly stroke. Additionally, clinical application of the study provided an objective and reliable evidence-based reference for the follow-up research, nursing staff, clinical workers, and health policy decision makers. However, there were some limitations. For one thing, some of the articles involved were of relatively low quality, with unclear randomization methods, allocation concealment, and uncalculated sample size, which affected the reliability of the conclusion in this study. For another, the fact that all the included studies are Chinese led to linguistic bias in the results. Therefore, follow-up studies should carefully be designed, implemented, and reported following the standard of RCTs [33]. Moreover, we found a moderate to high heterogeneity across our study, which might directly affect the reliability of our evidence. Although by implementing subgroup analyses based on the primary results, we identified the sources of heterogeneity caused by different treatment duration and regional development condition in different literature, there were still other hidden elements affecting the measured effect of PNS in this study. Consequently, it is crucial to carry out multi-center and larger-sample RCTs on treating elderly stroke patients with PNS so as to provide guidance for clinical treatment and follow-up research.

## 5. Conclusion

Our study found that PNS intervention or PNS combined with WM/TAU significantly improved the neurological function, DLAs, and the overall clinical efficacy in elderly stroke patients. Considering the quality of the included studies, the results of this study should be interpreted with caution. And it is necessary to conduct more multi-center RCTs with high quality to explore the efficacy and safety of PNS for elderly stroke patients in the future, thus contributing more reliable primary data to evidence-based decision-making.

## Figures and Tables

**Table 1:** Basic information on the included studies.

Number	Author	Date	Title
1	Ning Ao	2005	The effect of Xuesetong and Danshen on acute cerebral infarction
2	Yan Cao	2016	Effect of Xuesetong injection on neurological function and blood rheology in elderly ischemic stroke
3	Airong Zeng	2019	Clinical value of Xuesetong with alprostadil for cerebral infarction
4	Qingli Zeng	2015	Intervention effect of Xuesetong combined with edaravone on thrombosis in acute cerebral infarction
5	Dongmei Chang	2009	Xueshuantong combine with naloxone for the treatment of acute cerebral infarction
6	Guiying Che	2014	Study on the clinical efficacy of rehabilitation training with Xueshuantong in stroke patients
7	Zhenxian Shen	2015	The clinical effect of Xueshuantong combined with edaravone in treatment of acute cerebral infarction and its impact on neurological deficits and activities of daily living
8	Caihua Chen	2019	Efficacy of edaravone and Xuesetong in cerebral infarction
9	Hongfen Chen	2012	Evaluation of the treatment effect of acute cerebral infarction in Xueshuantong
10	Jie Chen	2022	Improvement effect of Xuesaitong injection on degree of neurological deficit in patients with cerebral infarction effect of panax notoginseng injection in the treatment of intracerebral hemorrhage
11	Lin Chen	2002	Clinical efficacy of naloxone and Xuesetong in elderly patients with acute cerebral infarction and its effect on neurological and motor function
12	Mai Chen	2018	Effect of Xuesaitong combined with nimodipine on cognitive function in patients with acute cerebral infarction
13	Minghui Chen	2019	Efficacy of sodium ozagrel and Xueshuantong in acute cerebral infarction
14	Mingxian Chen	2013	Clinical effect of Xueshuantong injection in acute cerebral infarction
15	Rui Chen	2005	Using Xuesetong for 22 patients with the acute phase of cerebral hemorrhage to improve circulatory treatment
16	Songlin Chen	2006	Clinical efficacy of Xuesetong and prostadil in the treatment of cerebral infarction
17	Sufen Chen	2018	Clinical effect of Xueshuantong freeze-dried powder injection in the treatment of acute cerebral infarction
18	Xishan Chen	2015	Clinical effect of Xuesetong injection in the treatment of 34 cases of acute cerebral infarction
19	Xiaoyan Chen	2009	Clinical effect of Xuesetong injection in the treatment of ischemic stroke
20	Xiaomei Chen	2015	Xueshuantong clinical observation on the treatment of the acute phase of ischemic stroke
21	Yuxun Chen	2013	Analysis of the efficacy of Xuesetong injection in patients with cerebral infarction
22	Huabi Chen	2014	Analysis of the efficacy of Xuesetong injection in patients with cerebral infarction observation of the result through treating patients with thrombotic cerebral infaration by Xie-Sai-Tong
23	Tiejun Dai	2005	Clinical observation on acute cerebral infarction treated with clopidogrel hydrochloride and Xueshuantong
24	Desheng Dan	2018	The effect of Xueshuantong and plasmin in acute cerebral infarction
25	Yu Deng	2013	Analysis of the curative effect of Xueshuantong freeze dried powder needle to cerebral infarction
26	Shuikang Ding	2008	Analysis of the curative effect of Xuesetong freeze dried powder needle to cerebral infarction
27	Shuikang Ding	2009	Clinical effect of benphthalein combined with Xuesetong in acute cerebral infarction
28	Haiyan Du	2018	Clinical observation on the curative effect of acute ischemic stroke treated with Xueshuantong injection and argatroban
29	Aiqin Fan	2018	Effect of Xuesaitong on peripheral blood WBC count and serum content of S-100B protein in patients with acute cerebral infarction
30	Rong Fan	2010	Clinical study on Xueshuantong for treating acute cerebral infarction
31	Dongmei Fang	2014	Clinical study on Xuesaitong injection for treating cerebral infarction
32	Fang Fang	2014	Clinical study on astragale injection combined with Sanqi Panax Notoginseng for Injection for treating ischemic stroke
33	Conglian Feng	2011	Efficacy of Xueshuantong combined with limb rehabilitation in the treatment of patients with cerebral arterial thrombosis
34	Xiangyon gFeng	2015	Clinical study of Xueshuantong combined with aspirin in the treatment of elderly patients with acute cerebral infarction
35	Xuecai Feng	2020	Clinical effect of Xueshuantong combined with sodium ozagrel in the treatment of acute cerebral infarction and the effect on nerve function
36	Zhe Feng	2020	Clinical effect of Xueshuantong combined with sodium ozagrel in the treatment of acute cerebral infarction and the effect on nerve function
37	Biao Fu	2017	Effect of Xueshuantong combined with butylphthalide soft capsules on the ECG and nerve function in patients with acute cerebral infarction effect of atorvastatin calcium dispersible tablet combined with Xuesaitong injection on blood lipid,
38	WeiFu	2018	Hemorheology and neurological function in patients with acute cerebral infarction clinical study on Xuesaitong in the treatment of 47 cases of acute cerebral infarction
39	Hanbiao Gan	2009	Hemorheology and neurological function in patients with acute cerebral infarction clinical study on Xuesaitong in the treatment of 47 cases of acute cerebral infarction
40	Chaoqun Gao	2019	Effect of Xuesaitong injection combined with fibrinogenase injection in elderly patients with ischemic stroke
41	Chu Gao	2019	Effects of Xueshuantong capsule combined with Xuesaitong for injection in elderly patients with cerebral infarction
42	Dongyang Gao	2022	Effect of Xueshuantong capsule on cerebral infarction and hemorheology
43	Guili Gao	2007	Xuesaitong injection in the treatment of cerebral infarction controlled observation
44	Yan Sun	2012	Study on Xuesaitong soft capsule in the treatment of 45 cases of ischemic stroke in recovery period
45	Hua Lan	2012	Study on Xueshuantong capsule and acupuncture and massage combined with Western medicine for treating of limb functions of cerebral infarction
46	Wei Gao	2022	Clinical study on Xueshuantong injection in the treatment of 48 cases of acute cerebral infarction
47	Yi Gao	2011	Effects of total Panax notoginseng saponins on neurological function and complement 3 in patients with acute intracerebral hemorrhage
48	Ying Gao	2011	Clinical study on edaravone injection combined with Xuesaitong for treating cerebral infarction
49	Zhiqing Gao	2019	Clinical study on Xueshuantong in the treatment of acute cerebral infarction
50	Haiping Gu	2016	Clinical study on Xuesaitong injection combined with ozagrel sodium for treating acute cerebral infarction
51	Zhiyan Gu	2014	Effect of Xueshuantong injection combined with urokinase intravenous thrombolysis on neurological deficit, vWF, and hs-CRP
52	Yafen Guan	2019	Study on clinical effect of edaravone injection combined with Xuesaitong for treating cerebral infarction
53	Binqing Guo	2019	Clinical study on Xuesaitong soft capsule in the treatment of 40 cases of cerebral infarction
54	Yanping Guo	2005	Study on the effect of Xueshuantong injection combined with Folium Ginkgo tablet in the treatment of acute cerebral infarction
55	Leisen Han	2003	Clinical study on edaravone combined with Xueshuantong in the treatment of acute cerebral infarction
56	Lijuan Han	2018	Influence of Xuesaitong on serum C-reactive protein level in the treatment of acute cerebral infarction and its clinical value
57	Yan Han	2014	Study on the effect of Naloxone combined with Xuesaitong in the treatment of elderly patients with acute cerebral infarction
58	Guoxiang Hu	2013	Effects of Xuesaitong capsules combined with butylphthalide soft capsules on cerebral blood folw and prognosis in patients with cerebral infarction in recovery period
59	Haitao Hu	2021	prognosis in patients with cerebral infarction in recovery period
60	Yaozhong	2017	Clinical curative effect of Xuesaitong injection combined with edaravone in treatment of elderly patients with acute cerebral infarction and influence on hemorheology of patients with plasma C reactive protein clinical effect of Ureklin combined with Xueshuantong on patients with the clinical effect of Ureklin combined
61	Debo Huang	2019	With Xueshuantong on acute ischemic stroke and changes of neurological deficit score and ability of daily living were analyzed
62	Huibin Huang	2009	Therapeutic effect of Xueshuantong for acute cerebral infarction and its influence on hemodynamics Clinical study on 150 cases with acute cerebral infarction treated by Xueshuantong
63	Meilin Huang	2012	Study on the effect of compound Xueshuantong capsules in the treatment of ischemic stroke
64	Wubiao Huang	2018	Influence of notoginseng triterpeneson applied at early stage on plasma MMP-9 level and neurological function recovery in patients with cerebral hemorrhage
65	Lijun Jia	2009	Curative effect observation of Xuesaitong injection for cerebral infarction
66	Mingxia Jiang	2004	Clinical observation of 40 cases of cerebral thrombosis treated with Xueshuantong
67	Rongfeng Jiang	2012	Influence of Xueshuantong injection on serum hypersensitivity C-reactive protein and interleukin-6 levels in patients with acute cerebral infarction
68	Yan Jiao	2016	Study on the effect of Xueshuantong injection in the treatment of cerebral infarction
69	Bo Jin	2015	Observation of clinical effect for Xuesaitong injection in treatment 70 cases with cerebral infarction patient
70	Yunjing Jin	2002	Effect and the changes of neurological function of aspirin combined with saponins of Panax notoginseng in the treatment of cerebral infarction
71	Xulin Ke	2016	In accordance with the adr and blood plug joint for the treatment of cerebral infarction clinical curative effect analysis
72	Yinchun Leng	2016	Clinical study on Sanqi Tongshu combined with edaravone in the treatment of acute cerebral infarction
73	Chunhong Li	2012	Effect of edaravone combined with Xueshuantong injection on acute cerebral infarction
74	Cunming Li	2014	Observation on curative effects of treatment with Panax notoginsenoside injection combined with batroxobin for acutely cerebral infarction in 31 patients
75	Fei Li	1999	Study on the effect of Xueshuantong injection in the treatment of acute cerebral infarction
76	Hui Li	2004	Clinical effect observation of Xueshuantong combined with ligustrazine treating acute ischemic stroke of type obstruction of collaterals by blood stasis
77	Huijun Li	2015	Clinical study on the effect of Xueshuantong injection in adjuvant treatment of ischemic stroke
78	Qin Li	2014	Study on Shuxuening injection combined with Xuesaitong injection in the treatment of cerebral infarction in 90 cases Clinical study on Xueshuantong combined with Aspirin in the treatment of elderly patients with acute cerebral
79	Ruiqi Li	2012	Clinical study on Xueshuantong combined with aspirin in the treatment of elderly patients with acute cerebral infarction
80	Song Li	2016	Discussion on the clinical value of Naloxone combined with Xuesaitong in the treatment of elderly patients with cerebral infarction
81	Wei Li	2017	Clinical observation of Xuesaitong combined Lumbrokinase in treating acute cerebral infarction
82	Wenlong Li	2010	Clinical study on Sanqi Panax notoginseng for injection combined with sodium ozagrel in the treatment of acute cerebral infarction
83	Yongzhon gLi	2013	Clinical efficacy of neurological recovery of butylphthalide soft capsules and Xueshuantong in patients with acute cerebral infarction
84	Yuqing Li	2016	Study on the effect of Sanqi Tongshu capsules combined with Huoxuetongmai tablet in the treatment of ischemic stroke
85	Yuanjun Li	2018	Clinical study on Xuesaitong combined with Levamlodipine in the treatment of cerebral hemorrhage and hypertension
86	Yuanlin Li	2015	Influence of Xueshuantong injection combined with alteplase on neurological function and vascular endothelial function in patients with acute cerebral infarction
87	Zhongyin Li	2021	Clinical study on Xuesaitong injection combined with edaravone in the treatment of acute cerebral hemorrhage
88	Bixian Liang	2011	The injection Xueshuantong the treatment of acute cerebral infarction with clinical efficacy and safety observed
89	Haiqiu Liang	2012	Effect of alteplase intravenous thrombolysis combined with Xueshuantong soft capsule on nerve function and quality of life in patients with acute cerebral infarction
90	Huili Liang	2018	Clinical study on Xuesaitong in treating syndrome of static blood blocking collaterals in recovery period of cerebral hemorrhage
91	Junjun Liang	2018	Clinical study on edaravone combined with Xueshuantong in the treatment of acute cerebral infarction
92	Kui Lin	2012	Analysis of clinical effect of applying Xueshuantong in acute lacunar cerebral infarction
93	Fangyin Lin	2018	Curative effect of Xuesaitong combined with edaravone on patients with cerebral infarction
94	Baogui Liu	2019	Clinical study on 40 cases with cerebral infarction treated by Xueshuantong
95	Bin Liu	2010	Study on 64 cases with acute cerebral infarction treated by Sanqi Panax Notoginseng for injection
96	Hongyan Liu	2014	Xueshuantong combined with Aspirin in treatment of senile acute cerebral infarction for 45 cases
97	Huiying Liu	2016	Study on 120 cases with cerebral infarction treated by Xuesaitong combined with edaravone injection
98	Lili Liu	2017	Clinical study on 160 cases with cerebral infarction treated by Xuesaitong injection
99	Qingping Liu	2012	Observation on the Therapeutic effect of fibrinogenase injection combined with Xueshuantong in elderly
100	Xiaojun Liu	2017	Patients with ischemic stroke study on the effect of Xuesaitong injection combined with phentolamine mesylate for injeacute cerebral infarction in treating
101	Yanli Liu	2011	Effect of compound Xueshuantong capsule on clopidogrel resistance of acute cerebral infarction and nerve function
102	Yang Liu	2020	Function of the clinical effect of Xuesaitong soft capsule to treat lacunar infarction
103	Shulin Lu	2010	Study on the effect of Xueshuantong in elderly patients with acute cerebral infarction and its influence on serum D-dimer level
104	Jinqiang Lu	2020	Serum D-dimer level clinical study on Xuesaitong injection in the treatment of acute cerebral infarction in 56 cases
105	Qin Ma	2015	Xue Sai Tong injection plus routine therapy for acute cerebral infarction and the influence on plasma C-reactive protein
106	Xiangdo Ng Luo	2011	Clinical study on Xueshuantong injection in the treatment of acute cerebral infarction in 60 cases
107	Junhua Ma	2010	Curative effect discussion of Xue Shuan Tong injection combined with edaravone in the treatment of acute cerebral infarction in 64 cases
108	Yong Ma	2015	Cerebral infarction in 64 cases
109	Hua Mei	2011	Clinical evaluation of combining Xuesaitong with vinpocetine on acute cerebral infarction Clinical observation on effect of ginkgo leaf extract and dipyridamole injection combined with Sanqi Panax
110	Xiaoneng Mo	2008	Notoginseng injection on acute cerebral infarction patients study on Xueshuantong injection in the treatment of cerebral hemorrhage in recovery period in 184 cases
111	Qiaolin Ou	2006	Clinical study on Xuesaitong combined with cytidine disodium triphosphate in the treatment of acute cerebral infarction in 43 cases
112	Dongzhen Piao	2016	Clinical efficacy of naloxone combined with Xueshuantong injection in the treatment of cerebral infarction and its effect on neurological function and motor function
113	Yan Pan	2019	Study on the effect of Xueshuantong combined with edaravone in the treatment of elderly patients with acute cerebral infarction and its influence on blood rheology and nerve function
114	Qiubo Qiao	2021	Discussion on clinical value of naloxone combined with Xuesaitong in the treatment of elderly patients with cerebral infarction
115	Liyuan Qin	2016	Effect of Panax notoginseng saponins in the treatment of acute cerebral infarct Xueshuantong injection in the treatment of acute cerebral infarction
116	Liqiang Qu	2014	Xueshuantong injection in the treatment of acute cerebral infarction
117	Qibo Ran	2011	Effect of Xueshuantong combined with edaravone on patients with acute cerebral infarction
118	Lihong Rong	2021	Clinical study on Xueshuantong injection in the treatment of acute cerebral infarction in 93 cases
119	Zhi Shang	2011	Study on the effect of Xueshuantong injection in the treatment of patients with ischemic stroke
120	Xinyou Shu	2017	Clinical study on vinpocetine combined with Xueshuantong in the treatment of acute cerebral infarction
121	Shuyi Song	2008	Effect observation on Xueshuantong injection in the treatment of transient ischemic attack
122	Ke Su	2012	Observation on the efficacy of combined therapy of thrombus and aspirin in the treatment of elderly patients
123	Dan Sun	2015	With acute cerebral infarction
124	Haijiao Sun	2021	Xueshuantong combined with aspirin in the treatment of acute cerebral infarction and its influence on S-100*β*
125	Weihui Qin	2021	Clinical study on Xueshuantong injection in the treatment of cerebral infarction in 30 cases clinical study on Xueshuantong in the treatment of acute cerebral infarction
126	Yujun Qin	2001	Clinical study on Xueshuantong in the treatment of acute cerebral infarction
127	Feifei Tan	2021	Effect of Xueshuantong injection on the serum levels of inflammatory factors in patients with acute cerebral infarction
128	Wenlan Tan	2018	Clinical study on Xuesaitong injection in the treatment of cerebral infarction in 100 cases
129	Yuming Tan	2003	The Clinical effect of naloxone combined with Xuesaaitong in treating se-nile cerebral infarction
130	Bo Tang	2018	Clinical study on Xueshuantong injection in the treatment of primary intracerebral hemorrhage
131	Lanzhi Tong	2012	Study on clinical effect of Xueshuantong combined with piracetam in the treatment of acute cerebral infarction and its influence on serum TC, SOD, and hs-CRP levels
132	Xiaolin Tong	2021	Efficacy observation of Xueshuantongin the treatment of acute cerebral infarction
133	Yongshe Ng Wang	2011	Effects of Xueshuantong on serum inflammatory factor levels and intracranial hematoma in patients with acute cerebral hemorrhage
134	Chaofeng Wang	2019	Clinical study on Naloxone combined with Xueshuantong in the treatment of acute cerebral infarction
135	Feifei Wang	2013	Effect of Xueshuantong injection combined with clopidogrel sulfate on neural function and oxidative stress indexes in patients with progressive ischemic stroke
136	Hailin Wang	2021	Study on the clinical effect of Xuesaitong combined with alprostadil in treating cerebral infarction and its influence on activities of daily living
137	Mengjue Wang	2018	Effect and safety of Xueshuantong injection in the treatment of acute cerebral infarction
138	Shan Wang	2021	Evaluation of 3-butylphthalide combined with Xueshuantong in treating ischemic stroke
139	Tianpeng Wang	2018	Clinical study on edaravone combined with Xuesaitong in the treatment of cerebral infarction
140	Xia Wang	2015	Effect of Xueshitong combined with butylphthalide on serum fibulin-5, Bdnf, and S100B in aged patients with acute cerebral infarction
141	Xiaoyu Wang	2018	Study on the effect of minimally invasive operation combined with Qingkailing and Xuesaitong in the treatment of acute cerebral hemorrhage clinical observation of 65 cases with acute cerebral infarction treated by Xuesaitong injection
142	Xinmin Wang	2007	Clinical observation of 65 cases with acute cerebral infarction treated by Xuesaitong injection
143	Xunwen Wang	2010	Clinical effect and safety of sodium ozagrel combined with Xueshuantong in the treatment of acute cerebral
144	Yanfen Wang	2019	Study on the clinical effect and safety of Xueshuantong combined with aspirin in treating elderly patients with acute cerebral infarction C
145	Yang Wang	2017	Clinical effect of Fufang Xueshuantong capsules on elderly acute cerebral infarction and its impact on vascular endothelial cell function
146	Zhiqing Wang	2015	Influence of notoginseng triterpenes on serum C-reactive protein level in patients with acute cerebral infarction
147	Xianping Wei	2007	Clinical effect of sodium ozagrel combined with Xueshuantong in the treatment of acute cerebral infarction
148	Xiaohong Wei	2016	The clinical effect of Xuesaitong combined with alprostadil in the treatment of cerebral infarction
149	Yan Wen	2016	Study on the effect of Xueshuantong injection combined with acupuncture in recovery period of ischemia cerebral vessels disease
150	Chunfang Wu	2021	Clinical observation of 68 cases with acute cerebral infarction treated by Sanqitongshu capsules
151	Fangting Wu	2012	Study on the effect of Xuesaitong injection combined with compound Danshen injection in the treatment of acute cerebral infarction
152	Fen Wu	2014	Evaluation of clinical value of edaravone combined with Xueshuantong in the treatment of acute progressive cerebral infarction
153	Fenghua Wu	2019	Study on the effect of Xueshuantong injection in the treatment of cerebral infarction
154	Lijun Wu	2015	Clinical study on Xuesaitong soft capsule in treating syndrome of static blood blocking collaterals in recovery period of cerebral infarction
155	Qiongfen Wu	2011	Primary study on safety and clinical effect of Xuesaitong in the treatment of cerebral infarction
156	Shuhua Wu	2016	Observation of 30 cases with acute cerebral infarction treated by Naloxone combined with Xueshuantong
157	Xiaoqiu Wu	2001	Study on Xueshuantong injection combined with batroxobin in the treatment of acute cerebral infarction
158	Yu Wu	2013	Clinical study on Xuesaitong injection combined with cattle encephalon glycoside and ignotin injection in treatment of elderly patients with acute cerebral infarction
159	Zhigang Wu	2018	Clinical study on Xuesaitong combined with alprostadil in the treatment of cerebral infarction effect of Xuesaitong combined with butylphthalide injection on PARK7, GFAP, CXCL12, and sV
160	Dejun Xia	2018	Effect of Xuesaitong combined with butylphthalide injection on PARK7, GFAP, CXCL12, and sV-CAM-1 in patients with acute cerebral infarction
161	Jundong Xia	2019	Clinical study on clopidogrel sulfate combined with Xueshuantong in the treatment of acute cerebral infarction
162	Xiamixi Nuer	2020	Clinical effect of Xuesaitong combined with sodium ozagrel in the treatment of acute cerebral infarction
163	Yanfei Xiang	2015	Clinical effect and mechanism of Xuesaitong applied to cerebral infarction
164	Huiling Xie	2013	Clinical study of Xuesaitong combined with sodium ozagrel in the treatment of cerebral infarction
165	Shanyu Xiong	2013	The effects of xue-sai-tong injection combined with intravenous thrombolysis on hemodynamics, nerve function, serum Hcy, NSE, and S-100*β* in patients with ischemic stroke
166	Dengfu Yan	2020	Clinical effect of Xueshuantong combined with sodium ozagrel in the treatment of cerebral infarction
167	Hongjun Yang	2020	Clinical observation of 38 cases with acute cerebral infarction treated by Xuesaitong injection
168	Hua Yang	2005	Random parallel comparison research of Xuesaitong combined with Bayer aspirin randomized parallel controlled study
169	Junhua Yang	2017	Influence of Xueshuantong combined with butylphthalide soft capsule on neurological deficit score and quality of life in patients with cerebral infarction in recovery period
170	Shizhan Yang	2019	Clinical observation of Xueshuantong for 52 cases with acute cerebral infarction
171	Tao Yang	2010	Clinical research for 76 cases on acute cerebral infarction treated with Xueshuantong injection
172	Wenming Yang	2004	Curative effect of Xuesaitong injection on acute cerebral infarction of 50 patients study on the effect of naloxone combined with Xuesaitong in the treatment of elderly patients with cerebral
173	Deliang Yao	2007	Study on the effect of naloxone combined with Xuesaitong in the treatment of elderly patients with cerebral infarction
174	Li Yao	2021	Study on 76 cases with cerebral infarction treated by Xuesaitong
175	Xiaoli Yao	2013	Study on Xueshuantong injection in the treatment of progressive cerebral infarction Study on clinical effect of Xueshuantong combined with sodium ozagrel in the treatment of cerebral infarction
176	Xiaobin Ye	2008	Study on clinical effect of Xueshuantong combined with sodium ozagrel in the treatment of cerebral infarction in 80 cases and its influence on hemorheology effect of Xueshuanton
177	Jianjun Yin	2010	Effect of Xueshuantong combined with aspirin in the treatment of elderly patients with acute cerebral infarction
178	Hao Yu	2020	Clinical study on the effect of naloxone combined with Xuesaitong in the treatment of elderly patients with cerebral infarction
179	Qiang Yu	2016	Influence of fluoxetine hydrochloride combined with Sanqi Tongshu capsule on NIHSS score and serum CGRP and IGF-1 level in elderly patients with ischemic stroke
180	Weiping Zang	2021	Observation of the effect of Xueshuantong injection combined with ginkgo leaf extract in the treatment of cerebral infarction
181	Fenguo Zhang	2012	Study on Xuesaitong injection combined with edaravone in treating acute cerebral hemorrhage
182	Guosheng Zhang	2014	Clinical study on Xuesaitong combined with edaravone in treating cerebral infarction
183	Haijie Zhang	2013	Evaluation on the effect of Xuesaitong in the treatment of cerebral infarction
184	Hui Zhang	2017	Influence of Xueshuantong combined with piracetam on TCM syndrome score neurological function and serum NPY and Hcy in patients with cerebral ischemic stroke
185	Xiping Zhang	2021	Observation on 45 cases of progressive cerebral infarction treated by Batroxobin combined with Xueshuantong
186	Xiaoshi Zhang	2011	Observation on 45 cases of progressive cerebral infarction treated by batroxobin combined with Xueshuantong
187	Xiaoshi Zhang	2012	Clinical effect of Xueshuantong injection combined with early rehabilitation on patients with acute cerebral infarction
188	Yang Zhang	2014	Clinical study of sodium ozagrel combined with Xuesaitong in the treatment of acute cerebral infarction
189	Yinguang Zhang	2015	Study on clinical effect of Xuesaitong combined with edaravone in treating elderly patients with acute cerebral infarction and its influence on hemodynamics
190	Yingwei Zhang	2020	Observation on the effect of low molecular weight heparin combined with Xuesaitong in the treatment of acute cerebral infarction
191	Yongcheg Zhang	2006	Study on the effect of Xueshuantong combined with edaravone in treating elderly patients with acute cerebral infarction and its influence on cytokines, cerebral hemodynamics, and vascular endothelial function
192	Yuming Zhang	2018	Clinical observation of cerebral infarction treated by acupuncture combined with intravenous infusion of Xuesaitong
193	Yuqin Zhang	2010	Effect of Panax notoginseng saponins on serum NSE levels and functional recovery in patients with acute cerebral infarction
194	Chan Zhao	2016	Clinical observation on Xueshuantong injection in treatment of acute cerebral infarction patients with clopidogrel resistance
195	Jing Zhao	2015	Observation on the effect of Xuesaitong injection combined with early rehabilitation in the treatment of acute cerebral thrombosis
196	Kun Zhao	2019	Influence of early rehabilitation combined with Xuesaitong injection on quality of life in patients with cerebral infarction
197	Xian Zhao	2014	Observation of Xueshuantong injection in the treatment of cerebral thrombosis
198	Yanli Zhao	2012	Application of Xueshuantong injection in the treatment of cerebral infarction
199	Jianyong Zheng	2016	Study on clinical value of Xueshuantong injection in the treatment of ischemic stroke
200	Li Zheng	2015	Clinical value of protoparaxotril saporlirs combined with aspirin in the secondary prevention of cerebral infarction
201	Borong Zhou	2008	Clinical research of total saponin of Panax notoginseng in the treatment of early cerebral hemorrhage
202	Chunlin Zhou	2016	Study on the effect and safety of Xueshuantong injection in the treatment of acute cerebral infarction
203	Yudong Zhou	2015	Study on the effect of Xuesaitong in the treatment of cerebral infarction
204	Saihua Zhu	2012	Clinical observation of rehabilitation therapy combined with Xuesaitong in treating sequelae of cerebral infarction
205	Xiaomin Zhu	2018	Clinical observation of cerebral protective effect of Xuesaitong on patients with cerebral infarction
206	Rong Zou	2018	

**Table 2:** ROB individual (risk of bias of the included studies).

Publications	ROB items						
	Random sequence generation (Selection bias)	Allocation concealment (Selection bias)	Blinding of participants & personnel (Performance bias)	Blinding of outcome assessors (Detection bias)	Incomplete outcome data (Attrition bias)	Selective reporting (reporting bias)	Other bias
Fei Li, 1999	Low	Unclear	Unclear	Unclear	Low	High	Low
Yunjing Jin, 2002	Low	Unclear	Unclear	Unclear	Low	Low	Low
Mingxia Jiang, 2004	Low	Unclear	Unclear	Unclear	Low	Unclear	Low
Hua Yang, 2005	Low	Unclear	Unclear	Unclear	Low	Unclear	Low
Shuyi Song, 2008	Low	Low	Low	Unclear	Low	Low	Low
Borong Zhou, 2008	Low	Low	Unclear	Unclear	Low	Unclear	Low
Dongmei Chang, 2009	Low	Low	Unclear	Unclear	Low	Unclear	Low
Lijun Jia, 2008	Low	Low	Unclear	Unclear	Low	Unclear	Low
Ying Gao, 2011	Low	Low	Unclear	Unclear	Low	Unclear	Low
Xiangdong Luo, 2011	Low	Low	Unclear	Unclear	Low	Unclear	Low
Zhi Shang, 2011	Low	Low	Unclear	Unclear	Low	Low	Low
Hongfen Chen, 2012	Low	Unclear	Unclear	Unclear	Low	Unclear	Low
Haiqiu Liang, 2012	Low	Low	Unclear	Unclear	Low	Unclear	Low
Kui Lin, 2012	Low	Low	Unclear	Unclear	Low	Unclear	Low
Qiongfen Wu, 2012	Low	Unclear	Low	Low	Low	Unclear	Low
Guoxiang Hu, 2013	Low	Low	Unclear	Low	Low	Unclear	Low
Yan Han, 2014	Low	Low	Unclear	Unclear	Low	Unclear	Low
Guosheng Zhang, 2012	Low	Unclear	Unclear	Unclear	Low	Unclear	Low
Xiangyong Feng, 2015	Low	Unclear	Unclear	Unclear	Low	Unclear	Low
Yuanlin Li, 2015	Low	Unclear	Unclear	Unclear	Low	Unclear	Low
Zhiqing Wang, 2015	Low	Unclear	Unclear	Unclear	Low	High	Low
Jing Zhao, 2015	Low	Unclear	Unclear	Unclear	Low	Unclear	Low
Yan Cao, 2016	Low	Unclear	Unclear	Unclear	Low	High	Low
Xulin Ke, 2016	Low	Low	Unclear	Unclear	Low	Unclear	Low
Song Li, 2016	Low	Low	Unclear	Unclear	Low	Unclear	Low
Yuqing Li, 2016	Low	Low	Unclear	Unclear	Low	High	Low
Huiying Liu, 2016	Low	Unclear	Unclear	Unclear	Low	Unclear	Low
Yan Wen, 2016	Low	Unclear	Unclear	Unclear	Low	Unclear	Low
Qiang Yu, 2016	Low	Low	Unclear	Unclear	Low	Unclear	Unclear
Chunlin Zhou, 2016	Low	Unclear	Low	Unclear	Low	Unclear	Low
Yaozhong Hu, 2017	Low	Unclear	Unclear	Unclear	Low	Unclear	Low
Wei Li, 2017	Low	Low	Unclear	Unclear	Low	Unclear	Unclear
Xiaojun Liu, 2017	Low	Unclear	Unclear	Unclear	Low	High	Low
Yang Wang, 2017	Low	Unclear	Unclear	Low	Low	Unclear	Unclear
Junhua Yang, 2017	Low	Low	Unclear	Unclear	Low	Low	Low
Mai Chen, 2018	Low	Low	Unclear	Low	Low	Unclear	Unclear
Zhiqing Gao, 2018	Low	Unclear	Unclear	Unclear	Low	Unclear	Low
Fangying Ling, 2018	Low	Unclear	Unclear	Unclear	Low	Unclear	Unclear
Bo Tang, 2018	Low	Low	Unclear	Unclear	Low	Unclear	Unclear
Xiaoyu Wang, 2018	Low	Low	Unclear	Unclear	Low	High	Low
Zhigang Wu, 2018	Low	Unclear	Unclear	Low	Low	Unclear	Low
Dejun Xia, 2018	Low	Low	Unclear	Unclear	Low	Unclear	Unclear
Yuming Zhang, 2018	Low	Low	Unclear	Unclear	Low	Unclear	Unclear
Xiaomin Zhu, 2018	Low	Unclear	Unclear	Unclear	Low	Unclear	Unclear
Chu Gao, 2019	Low	Unclear	Unclear	Unclear	Low	Unclear	Low
Yafen Guan, 2019	Low	Unclear	Unclear	Unclear	Low	Unclear	Low
Xianzhong Qi, 2018	Low	Low	Unclear	Low	Low	Unclear	Unclear
Jundong Xia, 2019	Low	Low	Unclear	Low	Low	Unclear	Low
Kun Zhao, 2019	Low	Low	Unclear	Unclear	Low	Unclear	Low
Xuecai Feng, 2020	Low	Unclear	Unclear	Low	Low	Unclear	Low
Yang Liu, 2020	Low	Unclear	Unclear	Low	Low	Unclear	Low
Jinqiang Lu, 2020	Low	Unclear	Unclear	Unclear	Low	Unclear	Low
Hao Yu, 2020	Low	Low	Unclear	Unclear	Low	Unclear	Low
Yingwei Zhang, 2020	Low	Low	Unclear	Low	Low	Unclear	Low
Haiyan Du, 2021	Low	Unclear	Low	Unclear	Low	Unclear	Unclear
Li Yao, 2021	Low	Low	Unclear	Unclear	Low	Unclear	Unclear
Weiping Zang, 2021	Low	Low	Unclear	Low	Low	Unclear	Low
Xiping Zhang, 2021	Low	Low	Unclear	Unclear	Low	Unclear	Low
Dongyang Gao, 2022	Low	Unclear	Unclear	Unclear	Low	Unclear	Low
Xiaoqiu Wu, 2001	Low	Unclear	Unclear	Unclear	Low	Unclear	Low
Lin Chen, 2002	Low	Unclear	Unclear	Low	Low	Unclear	Low
Leisen Han, 2003	Low	Unclear	Unclear	Unclear	Low	Unclear	Low
Yuming Tan, 2003	Low	Unclear	Low	Unclear	Low	Unclear	Low
Hui Li, 2004	Low	Unclear	Unclear	Low	Low	Unclear	Low
Wenming Yang, 2004	Low	Unclear	Unclear	Unclear	Low	High	Low
Ning Ao, 2005	Low	Unclear	Unclear	Unclear	Low	Unclear	Low
Rui Chen, 2005	Low	Unclear	Unclear	Unclear	Low	Unclear	Low
Tiejun Dai, 2005	Low	Unclear	Unclear	Low	Low	Unclear	Unclear
Yanping Guo, 2005	Low	Low	Unclear	Unclear	Low	Unclear	Low
Songlin Chen, 2006	Low	Unclear	Unclear	Unclear	Low	Unclear	Low
Qiaolin Ou, 2006	Low	Unclear	Low	Unclear	Low	Unclear	Low
Yongcheng Zhang, 2006	Low	Unclear	Unclear	Unclear	Low	Unclear	Low
Guili Gao, 2007	Low	Unclear	Unclear	Unclear	Low	Unclear	Low
Xinmin Wang, 2007	Low	Unclear	Unclear	Unclear	Low	Unclear	Low
Xianping Wei, 2007	Low	Unclear	Unclear	Unclear	Low	High	Low
Deliang Yao, 2007	Low	Unclear	Unclear	Low	Low	Unclear	Low
Shuikang Ding, 2008	Low	Low	Unclear	Low	Low	Unclear	Low
Xiaoneng Mo, 2008	Low	Unclear	Unclear	Low	Low	Unclear	Low
Xiaobin Ye, 2008	Low	Unclear	Unclear	Low	Low	Unclear	Low
Xiaoyan Chen, 2009	Low	Unclear	Unclear	Unclear	Low	Unclear	Low
Shuikang Ding, 2009	Low	Low	Unclear	Low	Low	Unclear	Low
Hanbiao Gan, 2009	Low	Unclear	Unclear	Unclear	Low	High	Low
Huibing Huang, 2009	Low	Unclear	Unclear	Unclear	Low	Unclear	Low
Rong Fan, 2010	Low	Unclear	Unclear	Unclear	Low	Unclear	Low
Yunxie Guo, 2010	Low	Unclear	Unclear	Unclear	Low	Unclear	Low
Wenlong Li, 2010	Low	Unclear	Unclear	Unclear	Low	Unclear	Low
Bin Liu, 2010	Low	Unclear	Unclear	Low	Low	Unclear	Low
Shulin Lu, 2010	Low	Unclear	Unclear	Unclear	Low	Unclear	Low
Junhua Ma, 2010	Low	Unclear	Unclear	Unclear	Low	High	Unclear
Xunwen Wang, 2010	Low	Unclear	Unclear	Unclear	Low	Unclear	Low
Tao Yang, 2010	Low	Unclear	Unclear	Unclear	Low	Unclear	Low
Jianjun Yin, 2010	Low	Unclear	Unclear	Unclear	Low	Unclear	Low
Yuqin Zhang, 2010	Low	Unclear	Unclear	Unclear	Low	Unclear	Low
Conglian Feng, 2011	Low	Unclear	Unclear	Unclear	Low	Unclear	Unclear
Yi Gao, 2011	Low	Unclear	Unclear	Unclear	Low	Unclear	Low
Bixian Liang, 2011	Low	Unclear	Unclear	Unclear	Low	Low	Unclear
Yanli Liu, 2011	Low	Unclear	Unclear	Low	Low	Unclear	Low
Hua Mei, 2011	Low	Unclear	Unclear	Unclear	Low	Unclear	Low
Qibo Ran, 2011	Low	Unclear	Low	Unclear	Low	Unclear	Low
Yongsheng Wang, 2011	Low	Unclear	Unclear	Unclear	Low	Unclear	Low
Xiaoshi Zhang, 2011	Low	Unclear	Unclear	Unclear	Low	High	Low
Guofeng Gao, 2012	Low	Unclear	Unclear	Unclear	Low	Unclear	Low
Linrong Gao, 2012	Low	Unclear	Unclear	Unclear	Low	Unclear	Low
Meilin Huang, 2012	Low	Unclear	Low	Low	Low	Unclear	Low
Rongfeng Jiang, 2012	Low	Unclear	Unclear	Unclear	Low	Unclear	Low
Chunhong Li, 2012	Low	Unclear	Unclear	Low	Low	Unclear	Low
Ruiqi Li, 2012	Low	Unclear	Unclear	Unclear	Low	Unclear	Low
Qingping Liu, 2012	Low	Unclear	Low	Unclear	Low	Unclear	Low
Ke Su, 2012	Low	Unclear	Unclear	Unclear	Low	Unclear	Low
Lanzhi Tong, 2012	Low	Unclear	Low	Unclear	Low	Unclear	Low
Fangting Wu, 2012	Low	Unclear	Unclear	Unclear	Low	Unclear	Low
Fenguo Zhang, 2012	Low	Unclear	Low	Unclear	Low	High	Unclear
Xiaoshi Zhang, 2012	Low	Unclear	Unclear	Low	Low	Unclear	Low
Yanli Zhao, 2012	Low	Unclear	Unclear	Unclear	Low	High	Unclear
Saihua Zhu, 2012	Low	Unclear	Unclear	Unclear	Low	High	Unclear
Mingxian Chen, 2013	Low	Unclear	Unclear	Unclear	Low	Unclear	Low
Yuxun Chen, 2013	Low	Unclear	Unclear	Unclear	Low	Unclear	Low
Yu Deng, 2013	Low	Low	Unclear	Unclear	Low	Unclear	Low
Yongzhong Li, 2013	Low	Unclear	Unclear	Unclear	Low	High	Low
Feifei Wang, 2013	Low	Unclear	Unclear	Unclear	Low	Unclear	Low
Yu Wu, 2013	Low	Unclear	Unclear	Unclear	Low	Unclear	Low
Huilin Xie, 2013	Low	Low	Unclear	Unclear	Low	Unclear	Low
Shanyu Xiong, 2013	Low	Low	Unclear	Unclear	Low	High	Unclear
Xiaoli Yao, 2013	Low	Unclear	Unclear	Unclear	Low	Unclear	Low
Haijie Zhang, 2013	Low	Unclear	Unclear	Unclear	Low	Unclear	Low
Guiying Che, 2014	Low	Unclear	Unclear	Low	Low	Unclear	Low
Zhengquan Chen, 2014	Low	Unclear	Unclear	Unclear	Low	Unclear	Low
Dongmei Fang, 2014	Low	Unclear	Unclear	Unclear	Low	Unclear	Low
Fang Fang, 2014	Low	Unclear	Unclear	Unclear	Low	Unclear	Low
Zhiyan Gu, 2014	Low	Unclear	Unclear	Unclear	Low	Unclear	Low
Cunming Li, 2014	Low	Low	Unclear	Unclear	Low	Low	Low
Qin Li, 2014	Low	Low	Unclear	Unclear	Low	Unclear	Low
Hongyan Liu, 2014	Low	Unclear	Unclear	Unclear	Low	Unclear	Low
LiQiang Qu, 2014	Low	Unclear	Unclear	Unclear	Low	Unclear	Low
Fen Wu, 2014	Low	Unclear	Unclear	Unclear	Low	Unclear	Low
Yang Zhang, 2014	Low	Low	Unclear	Low	Low	Unclear	Low
Xian Zhao, 2014	Low	Low	Unclear	Unclear	Low	Unclear	Low
Qingli Zeng, 2015	Low	Unclear	Unclear	Unclear	Low	Unclear	Low
Xishan Chen, 2015	Low	Low	Unclear	Unclear	Low	High	Low
Xiaomei Chen, 2015	Low	Unclear	Unclear	Unclear	Low	Unclear	Unclear
Bo Jin, 2015	Low	Unclear	Unclear	Unclear	Low	Unclear	Unclear
Huijun Li, 2015	Low	Unclear	Unclear	Unclear	Low	Unclear	Low
Jianwei Lv, 2015	Low	Unclear	Unclear	Unclear	Low	Unclear	Low
Yong Ma, 2015	Low	Unclear	Unclear	Unclear	Low	Unclear	Low
Zhenxian Shen, 2015	Low	Low	Unclear	Low	Low	Unclear	Low
Dan Sun, 2015	Low	Unclear	Low	Unclear	Low	Unclear	Low
Xia Wang, 2015	Low	Unclear	Unclear	Unclear	Low	High	Unclear
Lijun Wu, 2015	Low	Unclear	Unclear	Unclear	Low	Unclear	Low
Yanfei Xiang, 2015	Low	Unclear	Unclear	Unclear	Low	High	Unclear
Yinguang Zhang, 2015	Low	Unclear	Unclear	Unclear	Low	High	Low
Li Zheng, 2015	Low	Low	Unclear	Unclear	Low	Unclear	Low
Yudong Zhou, 2015	Low	Unclear	Unclear	Unclear	Low	Unclear	Low
Haiping Gu, 2016	Low	Unclear	Unclear	Unclear	Low	High	Low
Yan Jiao, 2016	Low	Low	Unclear	Low	Low	Unclear	Low
Yinchun Leng, 2016	Low	Unclear	Unclear	Unclear	Low	High	Low
Dongzhen Piao, 2016	Low	Unclear	Unclear	Unclear	Low	Unclear	Low
Liyuan Qin, 2016	Low	Unclear	Unclear	Unclear	Low	Unclear	Unclear
Xiaohong Wei, 2016	Low	Unclear	Unclear	Unclear	Low	Unclear	Low
Shuhua Wu, 2016	Low	Low	Unclear	Unclear	Low	Unclear	Unclear
Can Zhao, 2016	Low	Unclear	Unclear	Unclear	Low	High	Low
Jianyong Zheng, 2016	Low	Unclear	Unclear	Unclear	Low	Unclear	Low
Zhe Feng, 2017	Low	Unclear	Unclear	Unclear	Low	High	Low
Biao Fu, 2017	Low	Low	Unclear	Unclear	Low	Unclear	Low
Lili Liu, 2017	Low	Unclear	Low	Unclear	Low	Unclear	Low
Xinyou Shu, 2017	Low	Unclear	Low	Unclear	Low	Unclear	Low
Hui Zhang, 2017	Low	Unclear	Unclear	Low	Low	Unclear	Unclear
Sufen Chen, 2018	Low	Low	Unclear	Unclear	Low	Unclear	Low
Desheng Dan, 2018	Low	Unclear	Unclear	Low	Low	Unclear	Low
Aiqin Fan, 2018	Low	Low	Unclear	Unclear	Low	High	Low
Wei Fu, 2018	Low	Low	Unclear	Low	Low	Unclear	Low
Lijuan Han, 2018	Low	Unclear	Unclear	Unclear	Low	Unclear	Low
Wubiao Huang, 2018	Low	Unclear	Unclear	Unclear	Low	Low	Low
Yuanjun Li, 2018	Low	Unclear	Unclear	Unclear	Low	Unclear	Low
Mengjue Wang, 2018	Low	Low	Unclear	Unclear	Low	Unclear	Low
Tianpeng Wang, 2018	Low	Low	Unclear	Low	Low	Unclear	Unclear
Airong Zeng, 2019	Low	Low	Unclear	Unclear	Low	Unclear	Unclear
Chaoqun Gao, 2019	Low	Low	Unclear	Unclear	Low	Unclear	Low
Shizhan Yang, 2019	Low	Unclear	Low	Unclear	Low	Unclear	Low
Huili Liang, 2018	Low	Low	Unclear	Unclear	Low	Unclear	Low
Junjun Liang, 2018	Low	Low	Unclear	Unclear	Low	Unclear	Unclear
Wenlan Tan, 2018	Low	Low	Unclear	Unclear	Low	Unclear	Low
Rong Zou, 2018	Low	Unclear	Unclear	Unclear	Low	Unclear	Unclear
Caihua Chen, 2019	Low	Unclear	Unclear	Low	Low	Unclear	Unclear
Minghui Chen, 2019	Low	Low	Unclear	Low	Low	Unclear	Unclear
Binqing Guo, 2019	Low	Unclear	Unclear	Unclear	Low	Unclear	Unclear
Debo Huang, 2019	Low	Unclear	Unclear	Unclear	Low	High	Low
Baogui Liu, 2019	Low	Low	Unclear	Low	Low	Unclear	Low
Chaofeng Wang, 2019	Low	Unclear	Unclear	Unclear	Low	Unclear	Low
Fenghua Wu, 2019	Low	Unclear	Unclear	Unclear	Low	Unclear	Unclear
Xiamixinuer·Aihemaiti, 2020	Low	Unclear	Unclear	Unclear	Low	Unclear	Low
Dengfu Yan, 2020	Low	Low	Unclear	Low	Low	Unclear	Low
Hongjun Yang, 2020	Low	Unclear	Unclear	Low	Low	Unclear	Low
Haitao Hu, 2021	Low	Unclear	Unclear	Unclear	Low	Unclear	Low
Zhongyin Li, 2021	Low	Low	Unclear	Unclear	Low	Unclear	Low
Qiubo Qiao, 2021	Low	Unclear	Unclear	Unclear	Low	Unclear	Unclear
Lihong Rong, 2021	Low	Unclear	Unclear	Low	Low	Unclear	Low
Haijiao Sun, 2021	Low	Low	Unclear	Low	Low	Unclear	Low
Weihui Qin, 2021	Low	Unclear	Unclear	Unclear	Low	Unclear	Unclear
Feifei Tan, 2021	Low	Unclear	Unclear	Unclear	Low	High	Unclear
Xiaolin Tong, 2021	Low	Unclear	Unclear	Unclear	Low	Unclear	Low
Hailin Wang, 2021	Low	Unclear	Unclear	Unclear	Low	Unclear	Low
Shan Wang, 2021	Low	Unclear	Unclear	Low	Low	Unclear	Low
Yanfen Wang, 2021	Low	Unclear	Unclear	Low	Low	Unclear	Low
Chunfang Wu, 2021	Low	Low	Unclear	Unclear	Low	Unclear	Low
Jie Chen, 2022	Low	Low	Unclear	Unclear	Low	High	Low
Wei Gao, 2022	Low	Unclear	Unclear	Unclear	Low	Unclear	Low

**Table 3:** Certainty assessment of included studies in the meta-analysis.

Interventions	Certainty assessment							No. of patients		Effect size	Certainty	Importance
	No. of studies	Study design	Risk of bias	Inconsistency	Indirectness	Imprecision	Other considerations	Intervention group	Control group	Absolute (95% CI)		
PNS alone	Neurological status (NIHSS)											
	77	RCT	Serious^a^	Serious^b^	Not serious	Not serious	None	4,495	4,094	RR: 1.197 (1.229 lower to 1.165 higher)	Low	CRITICAL
	Total clinical efficacy											
	59	RCT	Serious^a^	Serious^b^	Not serious	Not serious	None	3,079	2,996	SMD: −0.826 (−0.707 lower to −0.946 higher)	Low	CRITICAL
	Daily living activities											
	11	RCT	Serious^a^	Serious^b^	Not serious	Serious^c^	None	419	420	RR: 1.675 (2.133 lower to 1.218 higher)	Low	CRITICAL

PNS combined with WM/TAU	Neurological status (NIHSS)											
	100	RCT	Serious^a^	Serious^b^	Not serious	Not serious	None	5,144	5,105	RR: 1.191 (1.217 lower to 1.165 higher)	Low	CRITICAL
	Total clinical efficacy											
	100	RCT	Serious^a^	Serious^b^	Not serious	Not serious	None	5,191	5,156	SMD: −1.142 (−0.990 lower to −1.295 higher)	Low	CRITICAL
	Daily living activities											
	44	RCT	Serious^a^	Serious^b^	Not serious	Not serious	None	2,271	2,237	RR: 1.034 (1.168 lower to 0.900 higher)	Low	CRITICAL

^a^All studies had a high risk of bias based on the ROB assessment; ^b^moderate to high heterogeneity was detected (*I*^2^: 42.8% to 92.1%); ^c^the total number of sample size is less than 1,000 as recommended.

**Table 4:** Main analysis and stratified analysis of the effect of panax notoginseng saponins for stroke among older population.

Interventions	Outcomes and subgroups	Number of studies	Number of patients		Pool effect size (95% CI)	*P *_Z_	Heterogeneity		Effects model
			EG	CG			*I * ^2^ (%)	*P *_*H*_	
PNS alone	Outcomes								
	NIHSS^*∗*^	59	3,079	2,966	−0.826 (−0.946 to −0.707)	<0.001	78.9	<0.001	Random
	Total clinical efficacy^*∗∗*^	77	4,495	4,094	1.197 (1.165 to 1.229)	<0.001	52.9	<0.001	Random
	ADL^*∗*^	11	419	420	1.675 (1.218 to 2.133)	<0.001	87.7	<0.001	Random
	Subgroups analyses based on the total clinical efficacy								
	Region								
	Overall	77	4,495	4,094	1.197 (1.165 to 1.229)	<0.001	52.9	<0.001	Random
	Develop regions	42	2,121	2,026	1.195 (1.155 to 1.237)	<0.001	37.9	0.008	Random
	Developing or undevelop regions	34	2,374	2,068	1.196 (1.150 to 1.245)	<0.001	60.6	<0.001	Random
	Publication year								
	Overall	77	4,495	4,094	1.197 (1.165 to 1.229)	<0.001	52.9	<0.001	Random
	Above and equal 2015	26	1,272	1,268	1.193 (1.151 to 1.236)	<0.001	11.9	0.0291	Random
	Below 2015	51	3,223	2,826	1.193 (1.153 to 1.234)	<0.001	59.9	<0.001	Random
	Total sample size								
	Overall	77	4,495	4,094	1.197 (1.165 to 1.229)	<0.001	52.9	<0.001	Random
	Above or equal 100	35	2,914	2,566	1.195 (1.155 to 1.237)	<0.001	37.9	0.008	Random
	Below 100	42	1,581	1,528	1.196 (1.150 to 1.245)	<0.001	60.6	<0.001	Random
	Male to female ratio								
	Overall	68	4,067	3,696	1.198 (1.165 to 1.232)	<0.001	54.1	<0.001	Random
	Above or equal 1	62	3,444	3,316	1.203 (1.168 to 1.239)	<0.001	54.6	0.047	Random
	Below 1	6	623	380	1.155 (1.045 to 1.276)	<0.001	55.6	<0.001	Random

PNS combined with WM/TAU	Outcomes								
	NIHSS^*∗*^	100	5,191	5,156	−1.142 (−1.295 to −0.990)	<0.001	92.1	<0.001	Random
	Total clinical efficacy^*∗∗*^	100	5,144	5,105	1.191 (1.165 to 1.217)	<0.001	42.8	<0.001	Random
	ADL^*∗*^	44	2,271	2,237	1.034 (0.900 to 1.168)	<0.001	77.8	<0.001	Random
	Subgroups analyses based on the total clinical efficacy								
	Region								
	Overall	100	5,144	5,105	1.191 (1.165 to 1.217)	<0.001	42.8	<0.001	Random
	Develop regions	40	1,939	1,952	1.199 (1.144 to 1.256)	<0.001	65.3	<0.001	Random
	Developing or undevelop regions	60	3,205	3,153	1.181 (1.158 to 1.205)	<0.001	0.0	0.553	Random
	Publication year								
	Overall	100	5,144	5,105	1.191 (1.165 to 1.217)	0.0049	42.8	<0.001	Random
	Above and equal 2015	64	3,355	3,373	1.188 (1.138 to 1.240)	<0.001	35.7	0.003	Random
	Below 2015	36	1,789	1,732	1.192 (1.163 to 1.222)	<0.001	53.2	<0.001	Random
	Total sample size								
	Overall	100	5,144	5,105	1.191 (1.165 to 1.217)	<0.001	42.8	<0.001	Random
	Above or equal 100	50	3,281	3,225	1.182 (1.144 to 1.221)	0.0078	60.8	<0.001	Random
	Below 100	50	1,863	1,880	1.195 (1.164 to 1.227)	<0.001	0.0	0.708	Random
	Male to female ratio								
	Overall	92	4,723	4,684	1.190 (1.162 to 1.218)	<0.001	44.8	<0.001	Random
	Above or equal 1	87	4,439	4,400	1.188 (1.159 to 1.216)	<0.001	46.2	<0.001	Random
	Below 1	5	284	284	1.220 (1.132 to 1.314)	<0.001	0.0	0.5	Random

^*∗*^Pool effect sizes were presented as standard mean differences (SMDs); ^*∗∗*^Pool effect sizes were presented as risk ratio (RR).

ADL, activities of daily living; CG, control group; EG, experimental group; NIHSS, National Institutes of Health Stroke Scale; PNS, Panax notoginseng saponins; TAU, treatment as usual; TCE, total clinical efficacy; WM, Western medicine.

## Data Availability

Some or all data generated or analyzed during this study are included in this published article or in the data repositories listed in references.

## Appendix

### A. PubMed

  #1 “Stroke” [Mesh]
   #2 (((((((((((((((((((((((((((Strokes[Title/Abstract]) OR (Cerebrovascular Accident[Title/Abstract])) OR (Cerebrovascular Accidents[Title/Abstract])) OR (CVA (Cerebrovascular Accident[Title/Abstract]))) OR (CVAs (Cerebrovascular Accident[Title/Abstract]))) OR (Cerebrovascular Apoplexy[Title/Abstract])) OR (Apoplexy, Cerebrovascular[Title/Abstract])) OR (Vascular Accident, Brain[Title/Abstract])) OR (Brain Vascular Accident[Title/Abstract])) OR (Brain Vascular Accidents[Title/Abstract])) OR (Vascular Accidents, Brain[Title/Abstract])) OR (Cerebrovascular Stroke[Title/Abstract])) OR (Cerebrovascular Strokes[Title/Abstract])) OR (Stroke, Cerebrovascular[Title/Abstract])) OR (Strokes, Cerebrovascular[Title/Abstract])) OR (Apoplexy[Title/Abstract])) OR (Cerebral Stroke[Title/Abstract])) OR (Cerebral Strokes[Title/Abstract])) OR (Stroke, Cerebral[Title/Abstract])) OR (Strokes, Cerebral[Title/Abstract])) OR (Stroke, Acute[Title/Abstract])) OR (Acute Stroke[Title/Abstract])) OR (Acute Strokes[Title/Abstract])) OR (Strokes, Acute[Title/Abstract])) OR (Cerebrovascular Accident, Acute[Title/Abstract])) OR (Acute Cerebrovascular Accident[Title/Abstract])) OR (Acute Cerebrovascular Accidents[Title/Abstract])) OR (Cerebrovascular Accidents, Acute[Title/Abstract])
   #3 (“Stroke” [Mesh]) OR ((((((((((((((((((((((((((((Strokes[Title/Abstract]) OR (Cerebrovascular Accident[Title/Abstract])) OR (Cerebrovascular Accidents[Title/Abstract])) OR (CVA (Cerebrovascular Accident[Title/Abstract]))) OR (CVAs (Cerebrovascular Accident[Title/Abstract]))) OR (Cerebrovascular Apoplexy[Title/Abstract])) OR (Apoplexy, Cerebrovascular[Title/Abstract])) OR (Vascular Accident, Brain[Title/Abstract])) OR (Brain Vascular Accident[Title/Abstract])) OR (Brain Vascular Accidents[Title/Abstract])) OR (Vascular Accidents, Brain[Title/Abstract])) OR (Cerebrovascular Stroke[Title/Abstract])) OR (Cerebrovascular Strokes[Title/Abstract])) OR (Stroke, Cerebrovascular[Title/Abstract])) OR (Strokes, Cerebrovascular[Title/Abstract])) OR (Apoplexy[Title/Abstract])) OR (Cerebral Stroke[Title/Abstract])) OR (Cerebral Strokes[Title/Abstract])) OR (Stroke, Cerebral[Title/Abstract])) OR (Strokes, Cerebral[Title/Abstract])) OR (Stroke, Acute[Title/Abstract])) OR (Acute Stroke[Title/Abstract])) OR (Acute Strokes[Title/Abstract])) OR (Strokes, Acute[Title/Abstract])) OR (Cerebrovascular Accident, Acute[Title/Abstract])) OR (Acute Cerebrovascular Accident[Title/Abstract])) OR (Acute Cerebrovascular Accidents[Title/Abstract])) OR (Cerebrovascular Accidents, Acute[Title/Abstract]))
   #4 “Ischemic Stroke” [Mesh]
   #5 (((((((((((((((((((((((Ischemic Strokes[Title/Abstract]) OR (Stroke, Ischemic[Title/Abstract])) OR (Ischemic Stroke[Title/Abstract])) OR (Ischemic Strokes[Title/Abstract])) OR (Stroke, Ischemic[Title/Abstract])) OR (Cryptogenic Ischemic Stroke[Title/Abstract])) OR (Cryptogenic Ischemic Strokes[Title/Abstract])) OR (Ischemic Stroke, Cryptogenic[Title/Abstract])) OR (Stroke, Cryptogenic Ischemic[Title/Abstract])) OR (Cryptogenic Stroke[Title/Abstract])) OR (Cryptogenic Strokes[Title/Abstract])) OR (Stroke, Cryptogenic[Title/Abstract])) OR (Cryptogenic Embolism Stroke[Title/Abstract])) OR (Cryptogenic Embolism Strokes[Title/Abstract])) OR (Embolism Stroke, Cryptogenic[Title/Abstract])) OR (Stroke, Cryptogenic Embolism[Title/Abstract])) OR (Wake-up Stroke[Title/Abstract])) OR (Stroke, Wake-up[Title/Abstract])) OR (Wake up Stroke[Title/Abstract])) OR (Wake-up Strokes[Title/Abstract])) OR (Acute Ischemic Stroke[Title/Abstract])) OR (Acute Ischemic Strokes[Title/Abstract])) OR (Ischemic Stroke, Acute[Title/Abstract])) OR (Stroke, Acute Ischemic[Title/Abstract])
   #6 (“Ischemic Stroke”[Mesh]) OR ((((((((((((((((((((((((Ischemic Strokes[Title/Abstract]) OR (Stroke, Ischemic[Title/Abstract])) OR (Ischemic Stroke[Title/Abstract])) OR (Ischemic Strokes[Title/Abstract])) OR (Stroke, Ischemic[Title/Abstract])) OR (Cryptogenic Ischemic Stroke[Title/Abstract])) OR (Cryptogenic Ischemic Strokes[Title/Abstract])) OR (Ischemic Stroke, Cryptogenic[Title/Abstract])) OR (Stroke, Cryptogenic Ischemic[Title/Abstract])) OR (Cryptogenic Stroke[Title/Abstract])) OR (Cryptogenic Strokes[Title/Abstract])) OR (Stroke, Cryptogenic[Title/Abstract])) OR (Cryptogenic Embolism Stroke[Title/Abstract])) OR (Cryptogenic Embolism Strokes[Title/Abstract])) OR (Embolism Stroke, Cryptogenic[Title/Abstract])) OR (Stroke, Cryptogenic Embolism[Title/Abstract])) OR (Wake-up Stroke[Title/Abstract])) OR (Stroke, Wake-up[Title/Abstract])) OR (Wake up Stroke[Title/Abstract])) OR (Wake-up Strokes[Title/Abstract])) OR (Acute Ischemic Stroke[Title/Abstract])) OR (Acute Ischemic Strokes[Title/Abstract])) OR (Ischemic Stroke, Acute[Title/Abstract])) OR (Stroke, Acute Ischemic[Title/Abstract]))
   #7 ((“Stroke”[Mesh]) OR ((((((((((((((((((((((((((((Strokes[Title/Abstract]) OR (Cerebrovascular Accident[Title/Abstract])) OR (Cerebrovascular Accidents[Title/Abstract])) OR (CVA (Cerebrovascular Accident[Title/Abstract]))) OR (CVAs (Cerebrovascular Accident[Title/Abstract]))) OR (Cerebrovascular Apoplexy[Title/Abstract])) OR (Apoplexy, Cerebrovascular[Title/Abstract])) OR (Vascular Accident, Brain[Title/Abstract])) OR (Brain Vascular Accident[Title/Abstract])) OR (Brain Vascular Accidents[Title/Abstract])) OR (Vascular Accidents, Brain[Title/Abstract])) OR (Cerebrovascular Stroke[Title/Abstract])) OR (Cerebrovascular Strokes[Title/Abstract])) OR (Stroke, Cerebrovascular[Title/Abstract])) OR (Strokes, Cerebrovascular[Title/Abstract])) OR (Apoplexy[Title/Abstract])) OR (Cerebral Stroke[Title/Abstract])) OR (Cerebral Strokes[Title/Abstract])) OR (Stroke, Cerebral[Title/Abstract])) OR (Strokes, Cerebral[Title/Abstract])) OR (Stroke, Acute[Title/Abstract])) OR (Acute Stroke[Title/Abstract])) OR (Acute Strokes[Title/Abstract])) OR (Strokes, Acute[Title/Abstract])) OR (Cerebrovascular Accident, Acute[Title/Abstract])) OR (Acute Cerebrovascular Accident[Title/Abstract])) OR (Acute Cerebrovascular Accidents[Title/Abstract])) OR (Cerebrovascular Accidents, Acute[Title/Abstract]))) OR ((“Ischemic Stroke”[Mesh]) OR ((((((((((((((((((((((((Ischemic Strokes[Title/Abstract]) OR (Stroke, Ischemic[Title/Abstract])) OR (Ischemic Stroke[Title/Abstract])) OR (Ischemic Strokes[Title/Abstract])) OR (Stroke, Ischemic[Title/Abstract])) OR (Cryptogenic Ischemic Stroke[Title/Abstract])) OR (Cryptogenic Ischemic Strokes[Title/Abstract])) OR (Ischemic Stroke, Cryptogenic[Title/Abstract])) OR (Stroke, Cryptogenic Ischemic[Title/Abstract])) OR (Cryptogenic Stroke[Title/Abstract])) OR (Cryptogenic Strokes[Title/Abstract])) OR (Stroke, Cryptogenic[Title/Abstract])) OR (Cryptogenic Embolism Stroke[Title/Abstract])) OR (Cryptogenic Embolism Strokes[Title/Abstract])) OR (Embolism Stroke, Cryptogenic[Title/Abstract])) OR (Stroke, Cryptogenic Embolism[Title/Abstract])) OR (Wake-up Stroke[Title/Abstract])) OR (Stroke, Wake-up[Title/Abstract])) OR (Wake up Stroke[Title/Abstract])) OR (Wake-up Strokes[Title/Abstract])) OR (Acute Ischemic Stroke[Title/Abstract])) OR (Acute Ischemic Strokes[Title/Abstract])) OR (Ischemic Stroke, Acute[Title/Abstract])) OR (Stroke, Acute Ischemic[Title/Abstract])))
   #8 “Panax notoginseng”[Mesh]
   #9 ((((((((((((((((((((((((((((((((((((Panax notoginsengs[Title/Abstract]) OR (notoginsengs, Panax[Title/Abstract])) OR (fufang xueshuantong[Title/Abstract])) OR (Xueshuantong[Title/Abstract])) OR (Xue shuan tong[Title/Abstract])) OR (xueshuangtong capsule[Title/Abstract])) OR (compound xueshuantong capsule[Title/Abstract])) OR (xuesaitong soft capsule[Title/Abstract])) OR (sanqi tongshu capsule[Title/Abstract])) OR (xueshuantong injection[Title/Abstract])) OR (notoginseng saponin[Title/Abstract])) OR (total notoginsenoside[Title/Abstract])) OR (panax notoginseng saponins[Title/Abstract])) OR (radix notoginseng[Title/Abstract])) OR (radix panax notoginseng[Title/Abstract])) OR (sanqi[Title/Abstract])) OR (san qi[Title/Abstract])) OR (san-qi[Title/Abstract])) OR (panax pseudoginseng[Title/Abstract])) OR (pseudoginseng[Title/Abstract])) OR (notoginseng[Title/Abstract])) OR (pseudo ginseng[Title/Abstract])) OR (pseudo-ginseng[Title/Abstract])) OR (tianqi[Title/Abstract])) OR (tian qi[Title/Abstract])) OR (xuesetong[Title/Abstract])) OR (xue se tong[Title/Abstract])) OR (xuesaitong[Title/Abstract])) OR (xue sai tong[Title/Abstract])) OR (lulutong[Title/Abstract])) OR (lu lu tong[Title/Abstract])) OR (zheng kang nao ming[Title/Abstract])) OR (nao ming[Title/Abstract])) OR (luo tai[Title/Abstract])) OR (xin nao tai[Title/Abstract])) OR (san qi tong shu[Title/Abstract]))
   #10 (“Panax notoginseng”[Mesh]) OR (((((((((((((((((((((((((((((((((((((Panax notoginsengs[Title/Abstract]) OR (notoginsengs, Panax[Title/Abstract])) OR (fufang xueshuantong[Title/Abstract])) OR (Xueshuantong[Title/Abstract])) OR (Xue shuan tong[Title/Abstract])) OR (xueshuangtong capsule[Title/Abstract])) OR (compound xueshuantong capsule[Title/Abstract])) OR (xuesaitong soft capsule[Title/Abstract])) OR (sanqi tongshu capsule[Title/Abstract])) OR (xueshuantong injection[Title/Abstract])) OR (notoginseng saponin[Title/Abstract])) OR (total notoginsenoside[Title/Abstract])) OR (panax notoginseng saponins[Title/Abstract])) OR (radix notoginseng[Title/Abstract])) OR (radix panax notoginseng[Title/Abstract])) OR (sanqi[Title/Abstract])) OR (san qi[Title/Abstract])) OR (san-qi[Title/Abstract])) OR (panax pseudoginseng[Title/Abstract])) OR (pseudoginseng[Title/Abstract])) OR (notoginseng[Title/Abstract])) OR (pseudo ginseng[Title/Abstract])) OR ( pseudo-ginseng[Title/Abstract])) OR (tianqi[Title/Abstract])) OR (tian qi[Title/Abstract])) OR (xuesetong[Title/Abstract])) OR (xue se tong[Title/Abstract])) OR (xuesaitong[Title/Abstract])) OR (xue sai tong[Title/Abstract])) OR (lulutong[Title/Abstract])) OR (lu lu tong[Title/Abstract])) OR (zheng kang nao ming[Title/Abstract])) OR (nao ming[Title/Abstract])) OR (luo tai[Title/Abstract])) OR (xin nao tai[Title/Abstract])) OR (san qi tong shu[Title/Abstract])))
   #11 (randomized controlled trial [pt] OR controlled clinical trial [pt] OR randomized [tiab] OR placebo [tiab] OR drug therapy [sh] OR randomly [tiab] OR trial [tiab] OR groups [tiab]) NOT (animals [mh] NOT humans [mh])
   #12 (((“Stroke”[Mesh]) OR ((((((((((((((((((((((((((((Strokes[Title/Abstract]) OR (Cerebrovascular Accident[Title/Abstract])) OR (Cerebrovascular Accidents[Title/Abstract])) OR (CVA (Cerebrovascular Accident[Title/Abstract]))) OR (CVAs (Cerebrovascular Accident[Title/Abstract]))) OR (Cerebrovascular Apoplexy[Title/Abstract])) OR (Apoplexy, Cerebrovascular[Title/Abstract])) OR (Vascular Accident, Brain[Title/Abstract])) OR (Brain Vascular Accident[Title/Abstract])) OR (Brain Vascular Accidents[Title/Abstract])) OR (Vascular Accidents, Brain[Title/Abstract])) OR (Cerebrovascular Stroke[Title/Abstract])) OR (Cerebrovascular Strokes[Title/Abstract])) OR (Stroke, Cerebrovascular[Title/Abstract])) OR (Strokes, Cerebrovascular[Title/Abstract])) OR (Apoplexy[Title/Abstract])) OR (Cerebral Stroke[Title/Abstract])) OR (Cerebral Strokes[Title/Abstract])) OR (Stroke, Cerebral[Title/Abstract])) OR (Strokes, Cerebral[Title/Abstract])) OR (Stroke, Acute[Title/Abstract])) OR (Acute Stroke[Title/Abstract])) OR (Acute Strokes[Title/Abstract])) OR (Strokes, Acute[Title/Abstract])) OR (Cerebrovascular Accident, Acute[Title/Abstract])) OR (Acute Cerebrovascular Accident[Title/Abstract])) OR (Acute Cerebrovascular Accidents[Title/Abstract])) OR (Cerebrovascular Accidents, Acute[Title/Abstract]))) OR ((“Ischemic Stroke”[Mesh]) OR ((((((((((((((((((((((((Ischemic Strokes[Title/Abstract]) OR (Stroke, Ischemic[Title/Abstract])) OR (Ischemic Stroke[Title/Abstract])) OR (Ischemic Strokes[Title/Abstract])) OR (Stroke, Ischemic[Title/Abstract])) OR (Cryptogenic Ischemic Stroke[Title/Abstract])) OR (Cryptogenic Ischemic Strokes[Title/Abstract])) OR (Ischemic Stroke, Cryptogenic[Title/Abstract])) OR (Stroke, Cryptogenic Ischemic[Title/Abstract])) OR (Cryptogenic Stroke[Title/Abstract])) OR (Cryptogenic Strokes[Title/Abstract])) OR (Stroke, Cryptogenic[Title/Abstract])) OR (Cryptogenic Embolism Stroke[Title/Abstract])) OR (Cryptogenic Embolism Strokes[Title/Abstract])) OR (Embolism Stroke, Cryptogenic[Title/Abstract])) OR (Stroke, Cryptogenic Embolism[Title/Abstract])) OR (Wake-up Stroke[Title/Abstract])) OR (Stroke, Wake-up[Title/Abstract])) OR (Wake up Stroke[Title/Abstract])) OR (Wake-up Strokes[Title/Abstract])) OR (Acute Ischemic Stroke[Title/Abstract])) OR (Acute Ischemic Strokes[Title/Abstract])) OR (Ischemic Stroke, Acute[Title/Abstract])) OR (Stroke, Acute Ischemic[Title/Abstract])))) AND ((“Panax notoginseng”[Mesh]) OR (((((((((((((((((((((((((((((((((((((Panax notoginsengs[Title/Abstract]) OR (notoginsengs, Panax[Title/Abstract])) OR (fufang xueshuantong[Title/Abstract])) OR (Xueshuantong[Title/Abstract])) OR (Xue shuan tong[Title/Abstract])) OR (xueshuangtong capsule[Title/Abstract])) OR (compound xueshuantong capsule[Title/Abstract])) OR (xuesaitong soft capsule[Title/Abstract])) OR (sanqi tongshu capsule[Title/Abstract])) OR (xueshuantong injection[Title/Abstract])) OR (notoginseng saponin[Title/Abstract])) OR (total notoginsenoside[Title/Abstract])) OR (panax notoginseng saponins[Title/Abstract])) OR (radix notoginseng[Title/Abstract])) OR (radix panax notoginseng[Title/Abstract])) OR (sanqi[Title/Abstract])) OR (san qi[Title/Abstract])) OR (san-qi[Title/Abstract])) OR (panax pseudoginseng[Title/Abstract])) OR (pseudoginseng[Title/Abstract])) OR (notoginseng[Title/Abstract])) OR (pseudo ginseng[Title/Abstract])) OR ( pseudo-ginseng[Title/Abstract])) OR (tianqi[Title/Abstract])) OR (tian qi[Title/Abstract])) OR (xuesetong[Title/Abstract])) OR (xue se tong[Title/Abstract])) OR (xuesaitong[Title/Abstract])) OR (xue sai tong[Title/Abstract])) OR (lulutong[Title/Abstract])) OR (lu lu tong[Title/Abstract])) OR (zheng kang nao ming[Title/Abstract])) OR (nao ming[Title/Abstract])) OR (luo tai[Title/Abstract])) OR (xin nao tai[Title/Abstract])) OR (san qi tong shu[Title/Abstract]))))

### B. Embase

   #1 “cerebrovascular accident”/exp
   #2 “strokes”: ab, ti OR “cerebrovascular accident”: ab, ti OR “cerebrovascular accidents”: ab, ti OR “cva (cerebrovascular accident)”: ab, ti OR “cvas (cerebrovascular accident)”: ab, ti OR “cerebrovascular apoplexy”: ab, ti OR “apoplexy, cerebrovascular”: ab, ti OR “vascular accident, brain”: ab, ti OR “brain vascular accident”: ab, ti OR “brain vascular accidents”: ab, ti OR “vascular accidents, brain”: ab, ti OR “stroke, cerebrovascular”: ab, ti OR “cerebrovascular strokes”: ab, ti OR “apoplexy”: ab, ti OR “strokes, cerebrovascular”: ab, ti OR “cerebral strokes”: ab, ti OR “cerebral stroke”: ab, ti OR “stroke, cerebral”: ab, ti OR “strokes, cerebral”: ab, ti OR “stroke, acute”: ab, ti OR “acute stroke”: ab, ti OR “acute strokes”: ab, ti OR “strokes, acute”: ab, ti OR “cerebrovascular accident, acute”: ab, ti OR “acute cerebrovascular accident”: ab, ti OR “acute cerebrovascular accidents”: ab, ti OR “cerebrovascular accidents, acute”: ab, ti
   #3 “cerebrovascular accident”/exp
   #4 “ischemic strokes”: ti, ab OR “stroke, ischemic”: ab, ti OR “ischemic stroke”: ab, ti OR “ischemic strokes”: ab, ti OR “stroke, ischemic”: ab, ti OR “cryptogenic ischemic stroke”: ab, ti OR “cryptogenic ischemic strokes”: ab, ti OR “ischemic stroke, cryptogenic”: ab, ti OR “stroke, cryptogenic ischemic”: ab, ti OR “scryptogenic stroke”: ab, ti OR “cryptogenic strokes”: ab, ti OR “stroke, cryptogenic”: ab, ti OR “cryptogenic embolism stroke”: ab, ti OR “cryptogenic embolism strokes”: ab, ti OR “embolism stroke, cryptogenic”: ab, ti OR “stroke, cryptogenic embolism”: ab, ti OR “wake-up stroke”: ab, ti OR “stroke, wake-up: ab, ti OR “wake up stroke”: ab, ti OR “wake-up strokes”: ab, ti OR “acute ischemic stroke”: ab, ti OR “acute ischemic strokes”: ab, ti OR “ischemic stroke, acute”: ab, ti OR “stroke, acute ischemic”: ab, ti OR
   #5 “panax notoginseng”/exp OR “panax notoginseng extract”/exp
   #6 “panax notoginsengs”: ab, ti OR notoginsengs, panax”: ab, ti OR “fufang xueshuantong”: ab, ti OR “xueshuantong”: ab, ti OR “xue shuan tong”: ab, ti OR “xueshuangtong capsule”: ab, ti OR “ompound xueshuantong capsule”: ab, ti OR “xuesaitong soft capsule”: ab, ti OR “tsanqi tongshu capsule”: ab, ti OR “xueshuantong injection”: ab, ti OR “notoginseng saponin”: ab, ti OR “total notoginsenoside”: ab, ti OR “panax notoginseng saponins”: ab, ti OR “radix notoginseng”: ab, ti OR “radix panax notoginseng”: ab, ti OR “sanqi”: ab, ti OR “san qi”: ab, ti OR “san-qi”: ab, ti OR “panax pseudoginseng”: ab, ti OR “pseudoginseng”: ab, ti OR “notoginseng”: ab, ti OR “pseudo ginseng”: ab, ti OR “pseudo-ginseng”: ab, ti OR “tianqi”: ab, ti OR “tian qi”: ab, ti OR “xuesetong”: ab, ti OR “xue se tong”: ab, ti OR “xuesaitong”: ab, ti OR “xue sai tong”: ab, ti OR “lulutong”: ab, ti OR “lu lu tong”: ab, ti OR “zheng kang nao ming”: ab, ti OR “nao ming”: ab, ti OR “luo tai”: ab, ti OR “luotai”: ab, ti OR “xin nao tai”: ab, ti OR “xinnaotai”: ab, ti OR “naoming”: ab, ti OR “san qi tong shu”: ab, ti OR “sanqitongshu”: ab, ti OR
   #7 “crossover procedure”: de OR “double-blind procedure”: de OR “randomized controlled trial”: de OR “single-blind procedure”: de OR random^*∗*^: de, ab, ti OR factorial^*∗*^: de, ab, ti OR crossover^*∗*^: de, ab, ti OR ((cross NEXT/1over^*∗*^): de, ab, ti) OR placebo^*∗*^: de, ab, ti OR ((doubl^*∗*^NEAR/1 blind^*∗*^): de, ab, ti) OR ((singl^*∗*^ NEAR/1 blind^*∗*^): de, ab, ti) OR assign^*∗*^: de, ab, ti OR allocat^*∗*^: de, ab, ti OR volunteer^*∗*^: de, ab, ti
   #8 #1 OR #2 OR #3 OR #4
   #9 #5 OR #6
   #10 #8 AND #9 AND #10

### C. Cochrane Library

   #1 MeSH descriptor: [Stroke] explode all trees
   #2 (Strokes): ti, ab, kw OR (Cerebrovascular Accident): ti, ab, kw OR (Cerebrovascular Accidents): ti, ab, kw OR (CVA (cerebrovascular accident)): ti, ab, kw OR (CVAs (cerebrovascular accident)): ti, ab, kw OR (cerebrovascular apoplexy): ti, ab, kw OR (apoplexy, cerebrovascular): ti, ab, kw OR (vascular accident, brain): ti, ab, kw OR (brain vascular accident): ti, ab, kw OR (brain vascular accidents): ti, ab, kw OR (vascular accidents, brain): ti, ab, kw OR (cerebrovascular stroke): ti, ab, kw OR (cerebrovascular strokes): ti, ab, kw OR (stroke, cerebrovascular): ti, ab, kw OR (strokes, cerebrovascular): ti, ab, kw OR (apoplexy): ti, ab, kw OR (cerebral stroke): ti, ab, kw OR (cerebral strokes): ti, ab, kw OR (stroke, cerebral): ti, ab, kw OR (strokes, cerebral): ti, ab, kw OR (stroke, acute): ti, ab, kw OR (acute stroke): ti, ab, kw OR (acute strokes): ti, ab, kw OR (strokes, acute): ti, ab, kw OR (cerebrovascular accident, acute): ti, ab, kw OR (acute cerebrovascular accident): ti, ab, kw OR (acute cerebrovascular accidents): ti, ab, kw OR (cerebrovascular accidents, acute): ti, ab, kw OR (ischemic stroke): ti, ab, kw OR (ischemic strokes): ti, ab, kw OR (stroke, ischemic): ti, ab, kw OR (ischemic stroke): ti, ab, kw OR (ischemic strokes): ti, ab, kw OR (stroke, ischemic): ti, ab, kw OR (cryptogenic ischemic stroke): ti, ab, kw OR (cryptogenic ischemic strokes): ti, ab, kw OR (ischemic stroke, cryptogenic): ti, ab, kw OR (stroke, cryptogenic ischemic): ti, ab, kw OR (cryptogenic stroke): ti, ab, kw OR (cryptogenic strokes): ti, ab, kw OR (stroke, cryptogenic): ti, ab, kw OR (cryptogenic embolism stroke): ti, ab, kw OR (cryptogenic embolism strokes): ti, ab, kw OR (embolism stroke, cryptogenic): ti, ab, kw OR (stroke, cryptogenic embolism): ti, ab, kw OR (wake-up stroke): ti, ab, kw OR (stroke, wake-up): ti, ab, kw OR (wake up stroke): ti, ab, kw OR (wake-up strokes): ti, ab, kw OR (acute ischemic stroke): ti, ab, kw OR (acute ischemic strokes): ti, ab, kw OR (ischemic stroke, acute): ti, ab, kw OR (stroke, acute ischemic): ti, ab, kw
   #3 MeSH descriptor: [Ischemic Stroke] explode all trees
  #4 (notoginsengs, panax): ti, ab, kw OR (panax notoginsengs): ti, ab, kw OR (panax notoginseng extract): ti, ab, kw OR (xueshuantong): ti, ab, kw OR (fufang xueshuantong): ti, ab, kw OR (xue shuan tong): ti, ab, kw OR (xueshuangtong capsule): ti, ab, kw OR (compound xueshuantong capsule): ti, ab, kw OR (xuesaitong soft capsule): ti, ab, kw OR (sanqi tongshu capsule): ti, ab, kw OR (xueshuantong injection): ti, ab, kw OR (notoginseng saponin): ti, ab, kw OR (total notoginsenoside): ti, ab, kw OR (panax notoginseng saponins): ti, ab, kw OR (radix notoginseng): ti, ab, kw OR (radix panax notoginseng): ti, ab, kw OR (sanqi): ti, ab, kw OR (san qi): ti, ab, kw OR (san-qi): ti, ab, kw OR (panax pseudoginseng): ti, ab, kw OR (pseudoginseng): ti, ab, kw OR (notoginseng): ti, ab, kw OR (pseudo ginseng): ti, ab, kw OR (pseudo-ginseng): ti, ab, kw OR (tianqi): ti, ab, kw OR (tian qi): ti, ab, kw OR (xuesetong): ti, ab, kw OR (xue se tong): ti, ab, kw OR (xuesaitong): ti, ab, kw OR (xue sai tong): ti, ab, kw OR (lulutong): ti, ab, kw OR (lu lu tong): ti, ab, kw OR (zheng kang nao ming): ti, ab, kw OR (nao ming): ti, ab, kw OR (luo tai): ti, ab, kw OR (xin nao tai): ti, ab, kw OR (san qi tong shu): ti, ab, kw
  #5 #1 OR #2 OR #3
  #6 #4 AND #5

### D. Web of Science

  #1 TS = (stroke OR stroke^*∗*^ OR cerebrovascular accident OR cerebrovascular accident^*∗*^ OR CVA (cerebrovascular accident) OR CVAs (cerebrovascular accident) OR cerebrovascular apoplexy OR apoplexy, cerebrovascular OR vascular accident, brain OR brain vascular accident OR brain vascular accidents OR vascular accidents, brain OR cerebrovascular stroke OR cerebrovascular strokes OR stroke, cerebrovascular OR strokes, cerebrovascular OR apoplexy OR cerebral stroke OR cerebral strokes OR stroke, cerebral OR strokes, cerebral OR stroke, acute OR acute stroke OR acute strokes OR strokes, acute OR cerebrovascular accident, acute OR acute cerebrovascular accident OR acute cerebrovascular accidents OR cerebrovascular accidents, acute OR ischemic stroke OR ischemic strokes OR stroke, ischemic OR ischemic stroke OR ischemic strokes OR stroke, ischemic OR cryptogenic ischemic stroke OR cryptogenic ischemic strokes OR ischemic stroke, cryptogenic OR stroke, cryptogenic ischemic OR cryptogenic stroke OR cryptogenic strokes OR stroke, cryptogenic OR cryptogenic embolism stroke OR cryptogenic embolism strokes OR embolism stroke, cryptogenic OR stroke, cryptogenic embolism OR wake-up stroke OR stroke, wake-up OR wake up stroke OR wake-up strokes OR acute ischemic stroke OR acute ischemic strokes OR ischemic stroke, acute OR stroke, acute ischemic)
  #2 TS = (panax notoginseng OR panax notoginsengs OR panax notoginseng extract OR notoginsengs, panax OR fufang xueshuantong OR xueshuantong OR xue shuan tong OR xueshuangtong capsule OR compound xueshuantong capsule OR xuesaitong soft capsule OR sanqi tongshu capsule OR xueshuantong injection OR notoginseng saponin OR total notoginsenoside OR panax notoginseng saponins OR radix notoginseng OR radix panax notoginseng OR sanqi OR san qi OR san-qi OR panax pseudoginseng OR pseudoginseng OR notoginseng OR pseudo ginseng OR pseudo-ginseng OR tianqi OR tian qi OR xuesetong OR xue se tong OR xuesaitong OR xue sai tong OR lulutong OR lu lu tong OR zheng kang nao ming OR nao ming OR luo tai OR xin nao tai OR san qi tong shu)
  #3 TS = clinical trial^*∗*^ OR TS = research design OR TS = comparative stud^*∗*^ OR TS = evaluation stud^*∗*^ OR TS = controlled trial^*∗*^ OR TS = follow-up stud^*∗*^ OR TS = prospective stud^*∗*^ OR TS = random^*∗*^ OR TS = placebo^*∗*^ OR TS = (single blind^*∗*^) OR TS = (double blind^*∗*^)
  #4 #1 AND #2 AND #3

## Abbreviations

PNS: Panax notoginseng saponins
RCT: Randomized controlled trials
RR: Relative risk
CI: Confidence interval
SMD: Standard mean difference
WM: Western medicine
TAU: Treatment as usual
XC: Xueshuantong capsule
XSC: Xuesaitong soft capsule
STC: Sanqi Tongshu capsule
CXC: Compound Xueshuantong capsule
TCM: Traditional Chinese Medicine
NIHSS: National Institutes of Health Stroke Scale
PTS: Panaxtriol saponins
BMP: Mean blood pressure
CVR: Cerebrovascular resistance.

## Supplementary Materials

Supplementary Materials Supplementary PRISMA-2020 checklist [20].
